# Isolation of pathogenic *Leptospira* strains from naturally infected cattle in Uruguay reveals high serovar diversity, and uncovers a relevant risk for human leptospirosis

**DOI:** 10.1371/journal.pntd.0006694

**Published:** 2018-09-13

**Authors:** Leticia Zarantonelli, Alejandra Suanes, Paulina Meny, Florencia Buroni, Cecilia Nieves, Ximena Salaberry, Carolina Briano, Natalia Ashfield, Caroline Da Silva Silveira, Fernando Dutra, Cristina Easton, Martin Fraga, Federico Giannitti, Camila Hamond, Melissa Macías-Rioseco, Clara Menéndez, Alberto Mortola, Mathieu Picardeau, Jair Quintero, Cristina Ríos, Víctor Rodríguez, Agustín Romero, Gustavo Varela, Rodolfo Rivero, Felipe Schelotto, Franklin Riet-Correa, Alejandro Buschiazzo, on behalf of the Grupo de Trabajo Interinstitucional de Leptospirosis Consortium

**Affiliations:** 1 Laboratorio de Microbiología Molecular y Estructural, Institut Pasteur de Montevideo, Montevideo, Uruguay; 2 Unidad Mixta UMPI, Institut Pasteur de Montevideo + Instituto Nacional de Investigación Agropecuaria INIA, Montevideo, Uruguay; 3 Departamento de Bacteriología, División Laboratorios Veterinarios “Miguel C. Rubino” Sede Central, Ministerio de Ganadería, Agricultura y Pesca, Montevideo, Uruguay; 4 Departamento de Bacteriología y Virología, Instituto de Higiene, Facultad de Medicina, Universidad de la República, Montevideo, Uruguay; 5 División Laboratorios Veterinarios “Miguel C. Rubino” Laboratorio Regional Noroeste, Ministerio de Ganadería, Agricultura y Pesca, Paysandú, Uruguay; 6 División Laboratorios Veterinarios “Miguel C. Rubino” Laboratorio Regional Este, Ministerio de Ganadería, Agricultura y Pesca, Treinta y Tres, Uruguay; 7 Instituto Nacional de Investigación Agropecuaria INIA, Estación Experimental La Estanzuela, Colonia, Uruguay; 8 Unité de Biologie des Spirochètes, Institut Pasteur, Paris, France; 9 Joint International Unit « Integrative Microbiology of Zoonotic Agents » IMiZA, Institut Pasteur de Montevideo, Montevideo, Uruguay / Institut Pasteur, Paris, France; 10 Département de Microbiologie, Institut Pasteur, Paris, France; Medical College of Wisconsin, UNITED STATES

## Abstract

Leptospirosis is a neglected zoonosis with worldwide distribution. The causative agents are spirochete bacteria of the *Leptospira* genus, displaying huge diversity of serovars, the identity of which is critical for effective diagnosis and vaccination purposes. Among many other mammalian species, *Leptospira* infects cattle, eliciting acute signs in calves, and chronic disease in adult animals often leading to abortions. In South America, and including in Uruguay, beef and dairy export are leading sources of national income. Despite the importance of bovine health, food safety, and bovine-related dissemination of leptospirosis to humans, extremely limited information is available as to the identity of *Leptospira* species and serovars infecting cattle in Uruguay and the South American subcontinent. Here we report a multicentric 3-year study resulting in the isolation and detailed characterization of 40 strains of *Leptospira* spp. obtained from infected cattle. Combined serologic and molecular typing identified these isolates as *L*. *interrogans* serogroup Pomona serovar Kennewicki (20 strains), *L*. *interrogans* serogroup Canicola serovar Canicola (1 strain), *L*. *borgpetersenii* serogroup Sejroe serovar Hardjo (10 strains) and *L*. *noguchii* (9 strains). The latter showed remarkable phenotypic and genetic variability, belonging to 6 distinct serogroups, including 3 that did not react with a large panel of reference serogrouping antisera. Approximately 20% of cattle sampled in the field were found to be shedding pathogenic *Leptospira* in their urine, uncovering a threat for public health that is being largely neglected. The two *L*. *interrogans* serovars that we isolated from cattle displayed identical genetic signatures to those of human isolates that had previously been obtained from leptospirosis patients. This report of local *Leptospira* strains shall improve diagnostic tools and the understanding of leptospirosis epidemiology in South America. These strains could also be used as new components within bacterin vaccines to protect against the pathogenic *Leptospira* strains that are actually circulating, a direct measure to reduce the risk of human leptospirosis.

## Author summary

Several species of the genus *Leptospira* cause leptospirosis, a disease that is transmitted from animals to humans (zoonosis). Leptospirosis is the most extended zoonosis worldwide, with over a million human cases each year. *Leptospira* spp. infect a broad range of wildlife and domestic animals, including cattle. In several South American countries beef and dairy exports rank among the most important national income sources, explaining why in Uruguay cattle outnumber human population by a factor of 4. Yet, we did not know which *Leptospira* species and serovariants (serovars) circulate among Uruguayan cattle. Current serologic diagnostic methods and whole killed-cell vaccination approaches, critically depend on using the proper serovars, which are hugely variable in *Leptospira* spp. from different regions of the world. Through a multidisciplinary consortium effort, we now report the isolation and typing of 40 strains of pathogenic *Leptospira* spp. An unexpectedly large variation in terms of species and serovars was found. These data are extremely important: 1- to improve diagnostics by updating the available reference antigen panels; 2- to evaluate the efficacy of novel vaccines; and, 3- to implement efficacious bovine vaccination as a means of reducing the incidence of bovine and human leptospirosis.

## Introduction

Leptospirosis is a zoonotic disease of worldwide importance caused by pathogenic spirochetes belonging to the genus *Leptospira* [1]. It affects humans and a broad range of domestic animals and wildlife. In cattle, leptospirosis is an important cause of reproductive failure, including abortions and stillbirths [2]. Infected bovines also constitute an active reservoir for the spread of the zoonotic disease, especially for humans in direct contact with infected animals including veterinarians, abattoir and farm workers, hunters, as well as scientists handling laboratory animals or during fieldwork [3, 4]. Domestic and wild animals are important reservoirs in rural areas, unlike urban settings where rats play a major dissemination role [5, 6]. Human infection with *Leptospira* spp. results from direct exposure if the source of infection is animal tissue, body fluids or urine, and from indirect exposure if the source is environmental, such as soil or urine-contaminated water. While the disease is endemic in many countries, it often presents as epidemic outbreaks, causing severe, sometimes fatal disease in both humans and animals [7, 8].

Since the first systematic studies in 1960–1970, serologic studies in animals have repeatedly shown high prevalence of exposure to *Leptospira* in Uruguay, with individual seropositivity in the 25–50% range, and herd prevalence figures of 50–70% [9, 10]. Leptospirosis is considered as a re-emerging bovine disease in Uruguay since 1998 [10], after what stricter epidemiologic surveillance policies have been adopted by governmental agencies. Human leptospirosis has been included into the official list of diseases of mandatory notification. Leptospirosis in Uruguay is endemic, with limited epidemic outbreaks in rural areas. The annual incidence of human leptospirosis is estimated at 15 per 100,000 [11], with precise figures not determined due to under-reporting and extremely scarce systematic studies in southern Latin America of morbidity/mortality burden [7]. The human disease appears to be associated with bovine infection, as well as to rainfalls and floods [11], with recent isolation efforts revealing the presence of three *L*. *interrogans* serovars, two *L*. *kirschneri* and one *L*. *borgpetersenii* [12, 13].

Despite the relevance of bovine leptospirosis as a cause of bovine abortions and infertility in Uruguay, there have been no extensive studies on the actual identities of *Leptospira* species and serovars obtained from animals in the field. There are currently no repositories of autochthonous isolates available in the public domain, thus constraining vaccine companies to the use of foreign strains as vaccine antigens. Even though Hardjo serovars have been suspected for years to be involved in bovine infection cases [2, 14], to the best of our knowledge only four *L*. *interrogans* and two *L*. *borgpetersenii* isolates belonging to this serovar have been reported in South America [15–17] obtained in Brazil and Chile. An early study also reported six Hardjo isolates in Argentina, without distinguishing the species [18], and two isolates of *L*. *interrogans* Hardjo were also reported, one in sheep from Brazil [19] and one in cattle from Mexico [20]. We now report the first results of a multicentric effort, over the course of 3 years, aimed at isolating pathogenic *Leptospira* strains in Uruguay, from infected cattle in the field and at abattoirs. A detailed serologic and genetic characterization of such isolates uncovers a larger than expected variety of *Leptospira* species and serovars. These data will be instrumental for the design of better bacterin vaccines, as well as for improving diagnosis and epidemiologic studies in Uruguay and neighboring South American countries.

## Methods

### Ethics statement

Urine and blood sampling from cattle in the field were performed by professional veterinarians, respecting international recommendations for animal welfare, with approval granted by the Ethics Committee for the Use of Animals for Experimentation (Comisión de Etica en el Uso de Animales de Experimentación CEUA), DILAVE, Ministry of Livestock, Agriculture and Fishery (Ministerio de Ganaderia, Agricultura y Pesca MGAP), Uruguay, according to national law #18,611. Permission to take samples for the study was received from the animal owners and the abattoirs.

### Identification of herds suspected of leptospirosis, and field urine and blood sampling

Forty-eight herds from both dairy and beef farms were sampled in this study, during a 33-month period (Jan 2015-Sep 2017). Private veterinarians who suspected the disease sent the first samples to our laboratory at the Ministry of Livestock, Agriculture and Fishery. Following current protocols in Uruguay, serum samples from 12 animals from each suspected herd, were screened by the microscopic agglutination test (MAT) [21] for preexisting antibodies against *Leptospira* (S1 Table). Farm selection for subsequent sample collection prioritized those herds with presumptive diagnosis of leptospirosis (MAT titers ≥200 against ≥1 pathogenic *Leptospira* reference serogroups). Farms with recorded history of abortions, infertility or acute disease, were also prioritized. Selected farms were visited from January 2015 to September 2017, and individual blood and urine samples from 19 animals were collected (aiming for ≥1 seropositive animal with a 95% confidence interval, using a conservative seroprevalence figure of ≥15% on a reference population of 1000 individuals; seroprevalence estimates from background serologic data in Uruguay are actually higher; the number of individual animals to sample was calculated with the software WinEpi http://www.winepi.net). Due to logistic constraints, in a few cases the number of animals per herd was slightly higher, overall sampling a total of 963 individual animals. Individuals to be sampled in each farm were selected according to recorded history when available, prioritizing animals with clinical signs of acute disease (especially calves with rectal temperature ≥ 39.5°C, jaundice and/or hemoglobinuria), previous antibody titers ≥200 by MAT, and/or history of abortions or infertility. If less than 19 animals met the latter criteria, additional animals (heifers or adult cows) from the same herd were included to complete the required number. A questionnaire was distributed to farmers, gathering information about history of leptospirosis and recent vaccination (<12 months) in the farm.

Blood samples were collected by coccygeal venipuncture using 5 mL tubes with clot activator. Sera were then stored at -20°C. Intramuscular administration of diuretics (~150 mg furosemide, Furo R, Ripoll) and thorough genital organ cleansing (wiping with 70% ethanol) preceded urine collection from individual animals. Approximately 60 mL of midstream urine was collected in sterile 120 mL containers (Bioset, Medicplast).

Urine samples (100 μL) were inoculated in the field, immediately or within 2 h of sample collection (for the rationale, see first section of Results), in 5 mL Ellinghausen-McCullough-Johnson-Harris (EMJH) medium (prepared with Leptospira Medium Base EMJH [Gibco] and albumin BovoLep [Bovogen Biologicals PTY Ltd]), supplemented with 100 μg/mL 5-fluorouracil (5-FU; Sigma) [21], and transported at 4°C to the laboratory together with the corresponding blood/serum samples in Vacutainer tubes (Vacutainer, BD-NJ, USA). In the laboratory, two serial 1:50 dilutions were made from the first urine-inoculated tube, in 5 mL EMJH medium supplemented with 5-FU (EMJH/FU), and all three dilutions were incubated at 29°C. The remaining volume of urine samples was conserved at 4°C for subsequent *lipL32* gene amplification (see below). Sera were used to determine anti-*Leptospira* titers by MAT following reported procedures [21]. Routine MAT tests used the national guide of positivity cutoff at titers ≥200. For comparison of reference *vs* local strains as MAT antigens (S5 Table), sera from animals from which pathogenic *Leptospira* spp. were isolated (only from those herds with no recent vaccination history) were tested by serial two-fold dilutions [21] starting from 1:100. The local strains used for the latter MATs, were chosen to represent each of the different serogroups identified in this work (IP1506001, IP1605021, IP1611024, IP1611025, IP1512017, IP1703027, IP1711049 and IP1512011, according to the numbering scheme defined in Table 1).

**Table 1:** Identification of autochthonous *Leptospira* spp. isolates by combining serologic and molecular approaches.

Isolate number	Department	Source	Year of isolation	Species (by *rrs* sequence)	VNTR^c^ (repeats profile)	Serogrouping (by MAT)	Presumptive serovar (by *rrs* + VNTR + MAT)	*secY* (genotype)
IP1507003	Paysandú*	urine	2015	*L*. *interrogans*	4-1-10	Pomona	Kennewicki	A
IP1509008	Canelones**	urine	2015	*L*. *interrogans*	4-1-10	Pomona	Kennewicki	A
IP1509009	Canelones**	urine	2015	*L*. *interrogans*	5-1-10	Pomona	Kennewicki	A
IP1509010	Artigas***	urine	2015	*L*. *interrogans*	5-1-10	Pomona	Kennewicki	A
IP1512011	Paysandú*	urine	2015	*L*. *interrogans*	5-1-10	Pomona	Kennewicki	A
IP1512014	Artigas***	urine	2015	*L*. *interrogans*	5-1-10	Pomona	Kennewicki	A
IP1512015	Artigas***	urine	2015	*L*. *interrogans*	5-1-10	Pomona	Kennewicki	A
IP1512016	Artigas***	urine	2015	*L*. *interrogans*	4-1-10	Pomona	Kennewicki	A
IP1603018	Artigas***	urine	2015	*L*. *interrogans*	5-0-10	Pomona	Kennewicki	A
IP1609022	Artigas***	urine	2015	*L*. *interrogans*	5-1-10	Pomona	Kennewicki	A
IP1610023	Lavalleja	urine^a^	2016	*L*. *interrogans*	5-1-10	Pomona	Kennewicki	A
IP1611026	Paysandú****	urine	2016	*L*. *interrogans*	4-1-10	Pomona	Kennewicki	A
IP1703028	Paysandú	urine^a^	2016	*L*. *interrogans*	4-1-10	Pomona	Kennewicki	A
IP1703029	Paysandú	kidney^a^	2016	*L*. *interrogans*	4-1-10	Pomona	Kennewicki	A
IP1710039	Artigas*******	urine	2017	*L*. *interrogans*	4-1-10	Pomona	Kennewicki	A
IP1710040	Artigas*******	urine	2017	*L*. *interrogans*	4-1-10	Pomona	Kennewicki	A
IP1710043	Artigas*******	urine	2017	*L*. *interrogans*	4-1-10	Pomona	Kennewicki	A
IP1710044	Artigas*******	urine	2017	*L*. *interrogans*	4-1-10	Pomona	Kennewicki	A
IP1710045	Artigas*******	urine	2017	*L*. *interrogans*	4-1-10	Pomona	Kennewicki	A
IP1710047	Paysandú	urine	2017	*L*. *interrogans*	4-1-10	Pomona	Kennewicki	A
IP1710049	Treinta y Tres	kidney^b^	2017	*L*. *interrogans*	1-10-2	Canicola	Canicola	A
IP1506001	Canelones**	urine	2015	*L*. *borgpetersenii*	1-5-4	Sejroe	Hardjo	B
IP1509005	Salto*****	urine	2015	*L*. *borgpetersenii*	1-4-4	Sejroe	Hardjo	B
IP1509006	Salto*****	urine	2015	*L*. *borgpetersenii*	1-5-4	Sejroe	Hardjo	B
IP1512013	Salto*****	urine	2015	*L*. *borgpetersenii*	1-4-4	Sejroe	Hardjo	B
IP1605020	Canelones**	urine	2015	*L*. *borgpetersenii*	1-5-5	Sejroe	Hardjo	B
IP1704030	Treinta y Tres******	urine	2017	*L*. *borgpetersenii*	1-4-4	Sejroe	Hardjo	B
IP1704031	Treinta y Tres******	urine	2017	*L*. *borgpetersenii*	1-4-4	Sejroe	Hardjo	B
IP1708034	Soriano	urine	2017	*L*. *borgpetersenii*	1-5-4	Sejroe	Hardjo	B
IP1708036	San José	kidney^a^	2017	*L*. *borgpetersenii*	1-5-4	Sejroe	Hardjo	B
IP1709038	Cerro Largo	kidney^a^	2017	*L*. *borgpetersenii*	1-5-4	Sejroe	Hardjo	B
IP1512017	Florida	urine^b^	2015	*L*. *noguchii*	ND	NA	ND	C
IP1605021	Salto	urine	2016	*L*. *noguchii*	ND	Pyrogenes	ND	D
IP1611024	Artigas	urine	2016	*L*. *noguchii*	ND	Australis	ND	E
IP1611025	Paysandú****	urine	2016	*L*. *noguchii*	ND	Autumnalis	ND	D
IP1703027	Durazno	urine^a^	2016	*L*. *noguchii*	ND	NA	ND	F
IP1705032	Florida	urine	2017	*L*. *noguchii*	ND	Autumnalis	ND	F
IP1708035	Rocha	kidney^a^	2017	*L*. *noguchii*	ND	Autumnalis	ND	G
IP1709037	Cerro Largo	kidney^a^	2017	*L*. *noguchii*	ND	Autumnalis	ND	H
IP1712055	Paysandú	urine	2017	*L*. *noguchii*	ND	NA	ND	I

*, **, ***, ****, *****, ******: ****** isolates obtained from animals in the same farm (indicated with equal number of asterisks)

^a^: samples collected at abattoirs

^b^: samples from calves with clinical signs of acute leptospirosis

^c^: the number of repeats for the VNTR4, VNTR7 and VNTR10 alleles are reported for *L*. *interrogans*; whereas for *L*. *borgpetersenii*, they correspond to the VNTR10, VNTRLb4 and VNTRLb5 alleles

NA: no detectable agglutination against any of the 24 serogroup-specific antisera included in the reference panel

ND: not determined

### Urine and kidney samples from abattoirs

Random samples of urine (vesical puncture) and kidneys were obtained at 22 slaughterhouses that received animals from geographic regions throughout the country. No indications of reproductive failure nor of any other health problems were recorded for slaughtered animals. Due to pipeline logistics at slaughterhouses, kidneys and urine samples did not correspond to the same animal such that individual samples were treated as independent. Urine samples were immediately inoculated in EMJH/FU, according to the same protocol as with field samples. Kidneys were transported in 4°C-refrigerated boxes to the laboratory and processed on arrival, 2–6 hours after sampling. A fragment of approximately 10 g of tissue was placed in a funnel, surface-sterilized by dousing with alcohol and flamed with a Bunsen burner. The tissue was then placed in a sterile stomacher bag and 10 mL of phosphate-buffered saline (PBS) were aseptically added. After breaking the tissue down to a pulp in the stomacher machine, the obtained suspension was allowed to settle for 15 minutes, 250 μL of supernatant were drawn and inoculated in 5 mL EMJH/FU (called tube A). From tube A, 500 μL were transferred to a second 5 mL EMJH/FU tube (tube B), thus obtaining also a 10-fold diluted culture. Finally, a third culture was also prepared from each sample by directly inoculating 5 mL Fletcher medium with a small cylinder of kidney tissue obtained with a Pasteur pipette. All cultures were incubated at 29°C.

### Culture conditions, isolation and conservation of *Leptospira* strains

In order to define a precise protocol for culture inoculation in the field after urine collection, decreasing numbers of *L*. *borgpetersenii* serovar Hardjo strain Sponselee cells, ranging from 10^7^ to 1 bacterium, were incubated in 1 mL filter-sterilized bovine urine. After variable times, 100 μL urine were inoculated in 5 mL EMJH for culture, and bacterial growth weekly monitored under a dark-field microscope.

For isolations, *Leptospira* cultures were incubated at 29°C and observed under dark-field microscopy weekly for up to 6 months [21]. In case of contamination by other microorganisms, the cultures were filtrated through a 0.22 μm sterile syringe filter (Millipore Corporation, MA, USA) and sub-cultured in fresh EMJH media. As soon as spirochete-like bacteria grew in specific cultures, the presence of pathogenic *Leptospira* species was assessed by PCR amplification of the *lipL32* gene (see below). Once no contamination observed, PCR-confirmed cultures were sub-cultured in EMJH media without 5-FU until exponential growth phase. *Leptospira* spp. isolates were then conserved at ≥10^8^ cells/mL in EMJH with 2.5% of dimethyl sulfoxide (Sigma) and flash-cooled in liquid nitrogen.

### *lipL32* PCR in urine samples and positive cultures for *Leptospira*

The *lipL32* gene was chosen as a marker of pathogenic *Leptospira* species [22–24]. PCR amplification of *lipL32* was performed using purified DNA from 10 mL of bovine urine samples. The urine was centrifuged at 10,000 g for 15 min, the pellet rinsed once with PBS pH 7.4, and total DNA was extracted with the PureLink Genomic DNA MiniKit (Invitrogen). *lipL32* PCR-amplification was achieved using oligonucleotide primers *lipL32F* (5´-ATCTCCGTTGCACTCTTTGC-3´) and *lipL32R* (5´-ACCATCATCATCATCGTCCA-3´) [25]. The PCR was performed in 50 μL 10 mM Tris.HCl pH 8.4, 50 mM KCl, 1.5 mM MgCl_2_, 200 μM dNTPs, 0.25 mg/mL bovine serum albumin (Sigma), 2 μM oligonucleotide primers, 1 U Taq DNA polymerase (Invitrogen) and 5 μL template DNA. PCR cycling comprised 1 denaturation step (5 min at 95°C), 35 amplification cycles (each cycle 30 s at 94°C, 30 s at 58°C and 1 min at 72°C) and a final extension step (7 min at 72°C). PCR products were analyzed by agarose gel electrophoresis and ethidium bromide staining, seeking for the expected 474 bp amplicon. Bovine serum albumin (Sigma) was added in the PCR reaction mix, 0.25 mg/mL, greatly reducing sporadic inhibitory effects of certain urine samples on the amplification reaction. An internal control was always included to quantify this potential inhibition issue, by spiking analyzed samples with 40 ng of *L*. *borgpetersenii* DNA. Positive amplifications products were randomly chosen in a few field samples, and sequenced confirming specific amplification of *Leptospira* DNA.

This *lipL32* PCR procedure was also performed to rank bacterial cultures (prioritizing more careful follow-ups), after DNA purification from 1 mL of EMJH cultures where suspect spirochetes had been observed by dark-field microscopy.

### Determination of *Leptospira* species by PCR amplification and partial sequencing of the 16S ribosomal RNA gene

DNA from *Leptospira* spp. bovine and human isolates were purified from 1 mL of EMJH culture using the PureLink Genomic DNA MiniKit (Invitrogen). Primers *LeptoA* (5´- GGCGGCGCGTCTTAAACATG-3´) and *LeptoB* (5´- TTCCCCCCATTGAGCAAGATT-3´) were used to amplify the 5’-terminal 331 bp fragment of the 16S rRNA gene (*rrs*) as previously described [26]. The resulting amplicons were sequenced in both senses using internal primers *LeptoC* (Forward) (5´-CAAGTCAAGCGGAGTAGCA-3´) and *Rs4* (Reverse)(5´-TCTTAACTGCTGCCTCCCGT-3´). Sequence quality was verified with the Chromas software, and consensus sequences were defined using BioEdit. All *rrs* sequences were deposited in GenBank (S2 Table). Consensus sequences were then compared with available sequences in GenBank using BLAST.

### Multilocus variable-number tandem repeat analysis

Multilocus variable-number tandem repeat (VNTR) analyses were performed according to published methods [27] using five discriminatory markers for VNTR loci 4, 7, 10, Lb4 and Lb5. Purified DNA from each isolate was used to amplify the VNTR4, VNTR7 and VNTR10 loci in *L*. *interrogans*, and the VNTR10, VNTRLb4 and VNTRLb5 loci in *L*. *borgpetersenii*. The GelAnalyzer 2010a software (http://www.gelanalyzer.com) was used to analyze the ethidium bromide-stained agarose electrophoresis gels, in which PCR products were resolved in parallel to 100-bp DNA ladder (Thermo Scientific) as molecular weight marker. The number of repeats for each VNTR locus was determined as: number of repeats = [PCR product size(bp)—flanking region (bp)] / repeat unit length (bp).

### Partial *secY* gene sequencing and analysis

DNA from *Leptospira* spp. bovine and human isolates were purified from 1 mL of EMJH culture using the PureLink Genomic DNA MiniKit (Invitrogen). The *secY* gene was partially amplified by PCR with primers *SecYF* (5´-ATGCCGATCATTTTTGCTTC-3´) and *SecYR* (5´-CCGTCCCTTAATTTTAGACTTCTTC-3´) as described [28]. The resulting 549 bp amplicon was sequenced in both senses. Sequence quality was verified with the Chromas software, and consensus sequences were defined using BioEdit. All *secY* sequences were deposited in GenBank (S2 Table) and compared to those available in PubMed, MLST (https://pubmlst.org/leptospira) and PATRIC (https://www.patricbrc.org) [29] databases. The phylogenetic analyses based on *secY* sequences were performed with MEGA 6.0 software (www.megasoftware.net) using the neighbor-joining method. The evolutionary distances were computed using the Tamura-Nei method and are in the units of the number of base substitutions per site. The reliability of branches was validated by generating 1000 bootstrap replicates. Based on the analysis of sequence similarities, *secY* genotypes were assigned.

### Serotyping

To determine the serogroup of isolated *Leptospira* strains, MAT was used with a panel of serogroup-specific rabbit antisera, spanning 24 *Leptospira* serogroups (KIT Royal Tropical Institute, S3 Table), performed in microtiter plates, mixing equal volumes of viable leptospires with serial 2-fold dilutions of each rabbit antiserum. After 2 h incubation at 37°C, agglutination of bacteria was observed under dark-field microscopy. The strain’s serogroup was assigned according to the antiserum that gave highest agglutination titer. Based on the combination of results from both serogroup determination and molecular typing (*rrs* gene partial sequencing and VNTR analysis), a presumptive serovar was assigned to all isolates belonging to *L*. *interrogans*, and *L*. *borgpetersenii* species, as previously described [27].

## Results

### Bovine urine affects *Leptospira* viability

Initial attempts to isolate *Leptospira* strains from bovine urine samples were unsuccessful. The initial protocol was based on collecting the urine from all sampled animals, and then inoculating them into the tubes with culture media. We asked whether bacterial cell viability could be compromised due to exposure to urine over time. As a first approach to address this issue, the particularly fastidious *L*. *borgpetersenii* serovar Hardjo was chosen [30] to perform *in vitro* tests of viability kinetics in bovine urine. Indeed, a critical maximum time of exposure was defined at less than 2 h (S4 Table), above which subsequent isolation success rates decreased significantly. Although it cannot be ruled out that other serovars might behave differently, based on these observations, all urine samples were inoculated in the field within 2 h of collection, resulting in successful isolations.

### PCR screening of urine samples is key to prioritize culture follow-ups toward isolation

**Fig 1:**
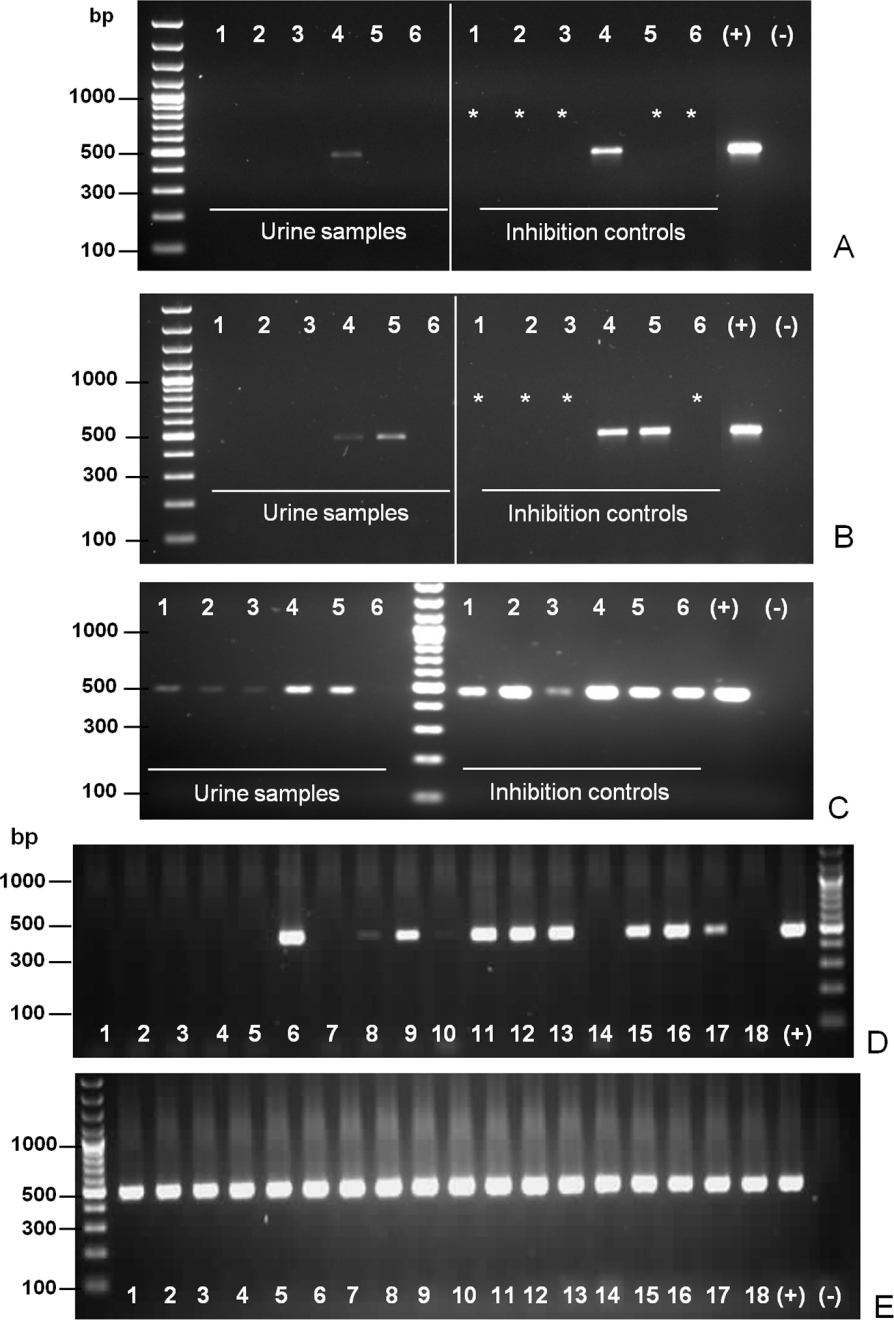
Screening of pathogenic *Leptospira* spp. in urine samples by PCR amplification of the *lipL32* gene. **(A)** PCR amplification of the *lipL32* gene, showing on the left side products obtained from 10 mL of urine without previous washing of the pellet, and on the right side the inhibition controls using pure DNA spiking. **(B)** Same as (A), except that the urine pellets on the left side were previously washed with PBS pH 7.4. **(C)** Same as (A) and (B), except that on the left side of the ladder urine pellets were previously washed with PBS pH 7.4 and BSA was included in the PCR mix. Asterisks show PCR reactions with total inhibition. **(D)** Typical *lipL32* amplification results, using optimized conditions as in (C), from randomly chosen urine samples collected in the field. **(E)** Corresponding inhibition controls for panel (D).

A second logistic challenge for isolation efforts from urine samples, was the high number of cultures subject to follow-up under dark-field microscopy. PCR amplification of *Leptospira lipL32* gene was optimized on bovine urine, eventually resulting in a robust method to prioritize cultures (Fig 1), identifying those samples that proved positive for pathogenic *Leptospira* spp. A strong inhibitory effect on *lipL32* PCR amplification was frequently observed, dependent on the urine sample (Fig 1A). This sample-dependent inhibition issue was solved by washing the bacterial pellet obtained after urine centrifugation with PBS pH 7.4 (Fig 1B), and then adding bovine serum albumin in the PCR mix (Fig 1C). The sensitivity of this PCR method was ≥100 *Leptospira* cells, estimated by spiking known amounts of bacteria to sterile urine samples. Specificity was assessed confirming a positive reaction with relevant serovars of pathogenic *Leptospira* species (*L*. *interrogans*, *L*. *noguchii*, *L*. *weilii*, *L*. *borgpetersenii* and *L*. *santarosai*), while undetectable with non-pathogenic *Leptospira* (*L*. *biflexa*) nor with unrelated species (*Escherichia coli*, *Pseudomonas aeruginosa*, *Salmonella* sp., *Staphylococcus aureus* and *Enterococcus* sp.).

Using this screening strategy, the presence of pathogenic *Leptospira* spp. DNA was confirmed in 193 urine samples, indicating that at least ~20% (193/963) of all studied animals were excreting pathogenic *Leptospira* in their urine (Fig 1D and 1E). False positive results from collected samples are highly unlikely, considering that *lipL32* is only present in the genomes of pathogenic *Leptospira* species [22], that no detectable amplification was observed with non-specific bacteria, and that randomly chosen amplicons from bovine urine samples confirmed 100% sequence identity with *Leptospira lipL32*. An environmental source of pathogenic bacteria during urine sample collection is highly unlikely as well, considering the sample collection procedure and the number of bacteria needed to attain the PCR sensitivity threshold. Following up with this approach at the herd level, 77% of the farms (37/48) that were studied, harbored ≥1 animal(s) excreting pathogenic *Leptospira*.

### Isolation of native strains of pathogenic *Leptospira* spp. infecting cattle

The sampling strategies, as detailed in Methods, were chosen to maximize the odds of isolating local strains of pathogenic *Leptospira* spp. from infected cattle. A two-pronged approach was followed: i- active and directed sampling in the field, at farms with suspicion of *Leptospira* infection; and, ii- random postmortem sampling of animals at slaughterhouses.

*Field sampling*. A total of 48 farms representing both beef and dairy cattle herds were visited from January 2015 to September 2017. They were distributed in 12 out of the 19 geographic departments in which the Uruguayan territory is divided. A total of 963 urine samples were collected and subjected to bacterial culture attempts and *lipL32* PCR screening. On average, *Leptospira* growth was detected by dark-field microscopy on cultures after 28 days (range 7–56 days).

Cultures that showed suspect bacteria, were subjected to *lipL32* PCR amplification, initially identifying 42 positive cultures from independent urine samples. Considering that 193 urine samples were positive by PCR screening, an estimated recovery rate of 21.7% (42/193) positive cultures from urine samples was achieved. From the original 42 positives, we ultimately obtained 32 pure cultures of *Leptospira* spp. (Table 1) from field animals, representing a 76.2% rate of success in isolating these bacteria from positive cultures, and a 3.3% global isolation success rate when considering the whole set of input urine samples (32/963). This latter figure should not be taken as a prevalence estimation of animals shedding leptospires (PCR-positive urine samples is a better indicator), since challenges in cultivating these fastidious bacteria are included in the global isolation rate.

*Sampling at abattoirs*. A total of 288 kidneys and 289 urine samples (representing 577 individual animals) were collected at slaughterhouses. According to the origin of slaughtered animals, all 19 departments of the country were included. 18 positive cultures of *Leptospira* were identified by dark-field microscopy and PCR amplification (*rrs* and *lipL32* genes), from which 8 isolates were eventually obtained, 3 from urine and 5 from kidney samples (Table 1).

### Identification of autochthonous pathogenic *Leptospira* strains

Overall, a total of 40 strains of pathogenic *Leptospira* were isolated from cattle along the course of this study, and characterized by combining serologic and molecular methods (Table 1). Recalling that initially 60 cultures had proved positive for *Leptospira* growth, the figures reveal that 20 could not be isolated (10 from field animals and 10 from slaughterhouses), due to overgrowth by contaminant species. Among the 40 characterized strains, 32 were isolated from live animals in the field (30 from cows or heifers, and 2 from calves with signs of acute leptospirosis), and 8 from adult carcasses at abattoirs (Table 1).

The *Leptospira* species were determined by PCR amplification and partial sequencing of the 16S rRNA gene (*rrs*). Three different pathogenic species were thus identified (Table 1): *L*. *interrogans* (n = 21), *L*. *borgpetersenii* (n = 10) and *L*. *noguchii* (n = 9).

Serogrouping of isolates was performed by MAT with a collection of 24 rabbit antisera against reference pathogenic serovars. All but one of the *L*. *interrogans* isolates corresponded to serogroup Pomona, the different one belonging to serogroup Canicola. The *L*. *borgpetersenii* strains all classed within serogroup Sejroe. In contrast, the *L*. *noguchii* isolates showed a broader variety of serogroups, including Pyrogenes (n = 1), Australis (n = 1), Autumnalis (n = 4), and 3 *L*. *noguchii* isolates that did not agglutinate with any of the reference antisera used.

Taking into account the identification of species and serogroup, together with the VNTR profiles (S1 Fig), it was possible to assign 20 *L*. *interrogans* strains to serovar Kennewicki, 1 *L*. *interrogans* to serovar Canicola, and the 10 *L*. *borgpetersenii* isolates to serovar Hardjo (Table 1). The serovars of the *L*. *noguchii* isolates could not be predicted, given that current VNTR profiling tables do not allow yet for serovar assignment of this species.

Twelve *L*. *interrogans*, five *L*. *borgpetersenii* and one *L*. *noguchii* strains, were isolated from farms with no history of vaccination (Table 2). Among such animals, MAT agglutination titers against reference strains were positive in ten cases (considering that national guidelines currently define less than 200 as non-reactive). However, when local isolates were added to the panel of MAT antigens for comparative purposes, 16 out of the 18 sera from non-vaccinated herds showed anti-*Leptospira* titers against the homologous autochthonous strain that was isolated (S5 Table). These results suggest that including local isolates of *Leptospira* spp. in the panel of antigens used for MAT may improve the sensitivity of the method. All the isolates recovered from herds with no history of vaccination, belonged to the homologous serogroup as shown by the seroreactivity data (S5 Table).

**Table 2:** MAT seroreactivity against reference *Leptospira* antigens and history of vaccination in cattle with positive culture of pathogenic *Leptospira* spp.

Strain #	Species identification	Serogroup / presumptive Serovar identification	Seroreactivity of the animal from which the isolate was obtained (serogroup/titer)	Seroreactivity of other animals in the same herd* (serogroup)	History of vaccination in the farm	Antigens included in the vaccine
IP1507003	*L*. *interrogans*	Pomona Kennewicki	Pomona / 200		No	
IP1509008	*L*. *interrogans*	Pomona Kennewicki	nr	Pomona	No	
IP1509009	*L*. *interrogans*	Pomona Kennewicki	Pomona / 400		No	
IP1509010	*L*. *interrogans*	Pomona Kennewicki	Pomona / 400		No	
IP1512011	*L*. *interrogans*	Pomona Kennewicki	nr	Pomona	No	
IP1512014	*L*. *interrogans*	Pomona Kennewicki	Pomona / 400		No	
IP1512015	*L*. *interrogans*	Pomona Kennewicki	Pomona / 6400		Yes (19 dpv)	*L*. *interrogans* serovars Pomona, Hardjo, Grippotyphosa, Icterohaemorrhagiae and Canicola
IP1512016	*L*. *interrogans*	Pomona Kennewicki	Pomona / 800		Yes (19 dpv)	*L*. *interrogans* serovars Pomona, Hardjo, Grippotyphosa, Icterohaemorrhagiae and Canicola
IP1603018	*L*. *interrogans*	Pomona Kennewicki	Pomona / 3200		Yes (19 dpv)	*L*. *interrogans* serovars Pomona, Hardjo, Grippotyphosa, Icterohaemorrhagiae and Canicola
IP1609022	*L*. *interrogans*	Pomona Kennewicki	Pomona / 1600		Yes (19 dpv)	*L*. *interrogans* serovars Pomona, Hardjo, Grippotyphosa, Icterohaemorrhagiae and Canicola
IP1611026	*L*. *interrogans*	Pomona Kennewicki	Pomona / 6400 Sejroe Hardjobovis / 1600 Sejroe Hardjoprajitno / 1600 Sejroe Wolffii / 800		Yes (26 dpv)	*L*. *interrogans* serovars Icterohaemorragiae, Pomona, Canicola, Wolffii, Hardjo, Tarassovi and Grippotyphosa *L*. *borgpetersenii* serovar Hardjo
IP1710039	*L*. *interrogans*	Pomona Kennewicki	Pomona / 6400		No	
IP1710040	*L*. *interrogans*	Pomona Kennewicki	Pomona / 6400 Sejroe Hardjobovis / 3200 Sejroe Hardjoprajitno / 1600 Sejroe Wolffii / 1600		No	
IP1710043	*L*. *interrogans*	Pomona Kennewicki	Pomona / 3200 Sejroe Hardjobovis / 800		No	
IP1710044	*L*. *interrogans*	Pomona Kennewicki	Pomona / 3200 Serjoe Hardjobovis / 3200 Serjoe Hardjoprajitno / 800		No	
IP1710045	*L*. *interrogans*	Pomona Kennewicki	Pomona / 6400		No	
IP1710047	*L*. *interrogans*	Pomona Kennewicki	nr	Sejroe Hardjobovis Sejroe Hardjoprjitno Sejroe Wolffii	No	
IP1506001	*L*. *borgpetersenii*	Sejroe Hardjo	Pomona / 400		No	
IP1509005	*L*. *borgpetersenii*	Sejroe Hardjo	nr	Sejroe Hardjobovis Sejroe Hardjoprjitno Sejroe Wolffii Pomona	No	
IP1509006	*L*. *borgpetersenii*	Sejroe Hardjo	nr	Sejroe Hardjobovis Sejroe Hardjoprjitno Sejroe Wolffii Pomona	No	
IP1512013	*L*. *borgpetersenii*	Sejroe Hardjo	nr	Sejroe Hardjobovis Sejroe Hardjoprjitno Sejroe Wolffii Pomona	No	na
IP1605020	*L*. *borgpetersenii*	Sejroe Hardjo	Sejroe Wolffii / 200		Y (120 dpv)	*L*. *interrogans* serovars Pomona, Hardjo, Grippotyphosa, Icterohaemorrhagiae and Canicola
IP1704030	*L*. *borgpetersenii*	Sejroe Hardjo	nr	Sejroe Hardjobovis, Sejroe Wolffii	Yes (nda)	nda
IP1704031	*L*. *borgpetersenii*	Sejroe Hardjo	nd	Sejroe Hardjobovis, Sejroe Wolffii	Yes (nda)	nda
IP1708034	*L*. *borgpetersenii*	Sejroe Hardjo	nr	Sejroe Hardjobovis Sejroe Hardjoprjitno Sejroe Wolffii	No	
IP1512017	*L*. *noguchii*	No agglutination^§^ / na^¶^	nr	nd	na	
IP1605021	*L*. *noguchii*	Pyrogenes	nr	Sejroe Hardjobovis Sejroe Hardjoprjitno Sejroe Wolffii	No	
IP1611024	*L*. *noguchii*	Australis / na^¶^	nr	Sejroe Hardjobovis Sejroe Hardjoprjitno Sejroe Wolffii Pomona	Yes (100 dpv)	*L*. *interrogans* serovars Icterohaemorragiae, Pomona, Canicola, Wolffii, Hardjo, Tarassovi and Grippotyphosa *L. borgpetersenii* serovar Hardjo
IP1611025	*L*. *noguchii*	Autumnalis / na^¶^	Sejroe Hardjobovis / 3200 Sejroe Hardjoprjitno / 3200 Sejroe Wolffii / 1600		Yes (26dpv)	*L*. *interrogans* serovars Icterohaemorragiae, Pomona, Canicola, Wolffii, Hardjo, Tarassovi and Grippotyphosa *L*. *borgpetersenii* serovar Hardjo
IP1705032	*L*. *noguchii*	Autumnalis / na^¶^	nr	Sejroe Hardjobovis Sejroe Wolffii Pomona	Yes (nda)	*L*. *interrogans* serovar Pomona
IP1712055	*L*. *noguchii*	No agglutination^§^ / na^¶^	nr	Pomona	Yes (150dpv)	*L*. *interrogans* serovars Icterohaemorragiae, Pomona, Canicola, Wolffii, Hardjo, Tarassovi and Grippotyphosa *L*. *borgpetersenii* serovar Hardjo

*Shown if the seroreactivity MAT titer <200 in the animal from which the isolate was recovered

**§** No agglutination against the reference panel of serogrouping antisera

**¶** No molecular proxy available for *L*. *noguchii* serovar assignment; **na**: not applicable; **nr**: non-reactive (below cutoff MAT titer 200); **nd**: not done; **nda**: no data available; **dpv**: days post vaccination when both urine and sera samples were collected

### Phylogeny of *Leptospira* isolates based on *secY* gene sequence analysis

**Fig 2:**
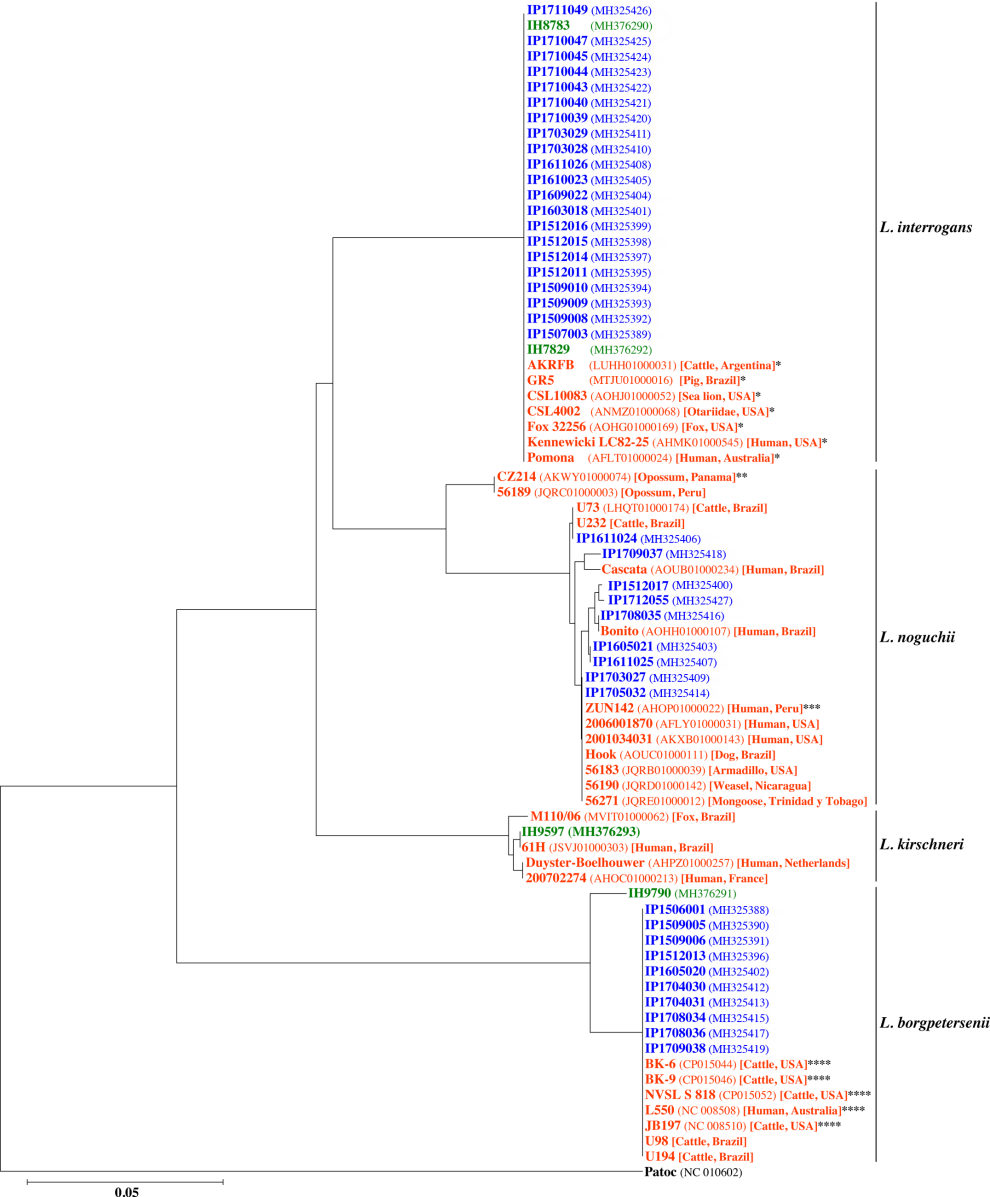
Phylogeny of *Leptospira* spp. isolates based on *secY* gene sequence analysis. Evolutionary history inferred by using the Neighbor-Joining method. The tree is drawn to scale, with branch lengths in the same units as those of the evolutionary distances used to infer the phylogenetic tree. The evolutionary distances were computed using the Tamura-Nei method and are in the units of the number of base substitutions per site. The analysis involved 76 partial sequences of the *secY* gene including the 40 bovine isolates from Uruguay that we are now reporting. Uruguayan strains from bovine hosts (in blue) and human patients (green) are compared to 32 additional sequences (in red) corresponding to isolates obtained elsewhere and from a variety of hosts, as indicated within brackets. Asterisks indicate the known serovar for isolates where such information is known, following the code: *serovar Pomona **serovar Panama ***serovar Autumnalis ****serovar Hardjo. Isolates obtained in Uruguay are named according to their strain denomination as "IP" (Institut Pasteur Montevideo) or "IH" (Instituto de Higiene) followed respectively by a 7- or a 4-digit number. GenBank accession numbers are indicated in parentheses. Well separated phylogenetic clades have a correspondence to different *Leptospira* species as indicated toward the right of the figure. The Patoc strain at the bottom of the panel belongs to the saprophytic species *L*. *biflexa*.

Genetic analysis of the 501bp *secY* allele was performed on the 40 typed isolates described in this work. Comparison to other *L*. *interrogans* (serovars Pomona and Canicola), *L*. *borgpetersenii* (serovar Hardjo) and *L*. *noguchii* sequences, obtained from other geographical regions and available in public databases, allowed to build a picture of related groups. Also included in this analysis were *secY* sequences obtained from 4 *Leptospira* strains recently isolated from human infections in Uruguay by one of the groups of our consortium [12, 13]. Such human isolates correspond to *L*. *interrogans*, *L*. *kirschneri* and *L*. *borgpetersenii* species. The dendrogram of partial *secY* sequence clustering, uncovered four phylogenetic clades that corresponded to genomospecies identified by partial *rrs* gene sequencing: *L*. *interrogans*, *L*. *borgpetersenii*, *L*. *kirschneri* and *L*. *noguchii* (Fig 2). The same 4-clades scenario emerged by calculating phylogeny with *rrs* gene sequences (S2 Fig). Only one homogeneous cluster was observed for the *L*. *interrogans secY* sequences, indicating that bovine isolates from Uruguay belonging to this species have close homology with isolates from South America (mainly from Brazil and Argentina) [31]. It is worth noting that two *L*. *interrogans* strains that had recently been isolated from human leptospirosis cases in Uruguay affecting rural workers [12, 13] clustered in the same *secY* clade together with the *L*. *interrogans* bovine isolates that we now describe. Concerning the *L*. *borgpetersenii* bovine strains, they also clustered with *L*. *borgpetersenii* serogroup Sejroe isolates from human and bovine sources in South America, Australia and USA; however, they showed no homology with the uruguayan *L*. *borgpetersenii* human isolate, which belongs to serogroup Ballum (F Schelotto, personal communication). Contrasting with such homogeneous clustering of *L*. *interrogans* and *L*. *borgpetersenii* strains, *secY* sequence analysis of the *L*. *noguchii* isolates revealed a substantially broader diversity, with isolates grouped in two distinct clusters. The first included two isolates, from Panama and Peru. The second cluster, with slight heterogeneity within, comprised all the *L*. *noguchii* isolates we are now reporting from Uruguay, as well as a number of other strains obtained from both human and animal origin in several countries of the American continent (Brazil, Nicaragua, Peru, Trinidad & Tobago, USA). Worth highlighting, the *secY* sequences of our bovine isolates IP1611024, IP1708035 and IP1709037, are identical to some of the *L*. *noguchii* strains recently reported in Brazil, isolated from cattle [32] and humans [33].

## Discussion

We are now reporting the isolation and typing of 40 native strains of pathogenic *Leptospira* spp. from infected cattle in Uruguay. This is the first systematic effort to isolate and type autochthonous *Leptospira* strains from cattle in this country, where bovine leptospirosis is a major concern as a cause of abortions and zoonotic dissemination. *L*. *interrogans* serovar Kennewicki (serogroup Pomona), our most frequent bovine isolate, has actually been also recovered from human patients with leptospirosis in Uruguay [12]. To further confirm this potential link between cattle and humans, we have now shown that the *secY* genotypes of both *L*. *interrogans* Kennewicki and Canicola serovars, are identical in *Leptospira* strains isolated from patients (rural workers) and from cattle (Fig 2), strongly suggesting that the latter disseminate the infection to exposed humans.

The successful culture of leptospires from bovine samples has likely been boosted by optimizing field sampling protocols, especially after quantifying time-dependent *Leptospira* viability in bovine urine. PCR screening has also been instrumental in prioritizing cultures, the number of which increased dramatically due to the systematic use of three culture dilutions per animal, themselves important to improve purity in some cases.

A total of 963 urine samples that were processed, eventually produced 42 positive cultures. Among these 42, 9 had produced negative PCR results at the time of urine sample screening. Two different scenarios explain such discrepancies: 8 of the 9 negative results, appeared early during our studies, and eventually proved to be the consequence of urine inhibition, triggering the optimization of our protocols (see Methods and Fig 1). Only in one sample we can strongly suggest that it is the PCR method’s sensitivity that explains the divergent result. In sum, *lipL32* PCR screening is an instrumental strategy to prioritize culture follow-ups, albeit not leading to discarding ongoing cultures. We are now optimizing a more sensitive real-time PCR approach, anticipated to also being more robust for screening purposes.

Regarding important, and frequently neglected factors that can lead to success or failure in nation-wide efforts based on field sampling, it is worth highlighting the voluntary participation of farmers and private veterinarians. Early arrangements ensuring for such implications were critical logistic factors for a swift sample collection strategy and for gathering useful information about herds and individual animals. Serial dilutions of the biologic samples on separate culture tubes were successfully used as a means to tackle contamination issues. Most of the positive cultures were successfully purified using the first two dilutions A and B, roughly 50% success from each one. Further diluting the inocula (tube C) allowed the recovery/purification of only 4 additional isolates. Overall, EMJH media outperformed Fletcher in our hands, with only two isolates grown from the latter that were also obtained with EMJH.

Combined serologic and molecular approaches revealed the presence of three different *Leptospira* species. Besides the anticipated *L*. *interrogans* and *L*. *borgpetersenii* species, known to be major infectious agents in cattle [2, 34], an important number of isolates corresponded to *L*. *noguchii*, both from field samples as well as from abattoirs. *L*. *noguchii* has been isolated from cattle in South America [14, 32, 33], but had never been reported in Uruguay, and extremely limited information is currently available about its epidemiologic importance. Are *L*. *noguchii* strains a relevant cause of acute disease or reproductive problems in cattle? One of the two strains that we have isolated from calves with signs of acute leptospirosis, was actually identified as *L*. *noguchii*, but more information is urgently needed in order to establish the contribution of this unanticipated species in the burden of veterinarian and human leptospirosis in South America. The other strain infecting a suspected acute case was confirmed as *L*. *interrogans* serogroup Canicola serovar Canicola, a highly virulent variant often isolated from dogs. Serovar Canicola is however not considered to be adapted to cattle, although it has been reported to infect bovine hosts incidentally, including recent reports in Brazil [35]. It is interesting to note that the isolates belonging to *L*. *interrogans* and *L*. *borgpetersenii*, displayed limited variation. The latter revealed a single VNTR profile (consistent with a single serovar, Hardjo, within the Sejroe serogroup), also coherent with a unique *secY* genotype (B). As for the *L*. *interrogans* strains, once again quite homogeneous features were found for all isolates, with 20 out of 21 compatible with serovar Kennewicki (serogroup Pomona), and displaying a single *secY* genotype (A). Only one *L*. *interrogans* was different, VNTR clearly matching the one expected for serovar Canicola (in line with Canicola serogroup sero-agglutination), yet sharing the same *secY* genotype A as the Pomona Kennewicki strains. In stark contrast, the 9 *L*. *noguchii* isolates uncovered an unexpected variety of serogroups. We have not yet assigned serovar types to these *L*. *noguchii* strains, given that the VNTR multilocus analysis scheme has not been validated for this *Leptospira* species on the basis of cross-agglutinin absorption tests (CAAT) with serovar-specific antisera. We are currently sequencing the whole genomes for all isolates and actively pursuing direct serovar identification by CAAT for the *L*. *noguchii* strains. However, it can immediately be recognized that all nine *L*. *noguchii* strains likely correspond to 9 distinct serovars, combining the information of serogrouping and *secY* genotypes. Three of them did not agglutinate with any of the reference antisera tested, which span 24 serogroups that cover major pathogenic *Leptospira* [36]. The other six corresponded to serogroups Pyrogenes, Australis and Autumnalis, the latter including four different isolates, all of which differed in *secY* genotypes (D, F, G and H). The three *L*. *noguchii* isolates that did not react with serogroup-specific reference antisera, revealed as yet three additional *secY* genotypes (C, F and I), hence likely pertaining to three disparate serovars as well.

Serogroup Pomona is one of the most common variants isolated from animals worldwide [37]. This serogroup displays important genetic diversity, as revealed by restriction endonuclease analysis (REA) [38], even within serovars. However, the REA-based genetic profiles of Pomona serovar Kennewicki, show high stability among isolates from a single outbreak [39] and, interestingly, a strong correlation between specific hosts and corresponding REA profile. Those results are consistent with our study: analyzed by *secY* allele genotyping, a high homogeneity was observed in all Pomona Kennewicki isolates from cattle, despite the broad geographic distribution of the isolates, including those obtained in the field and from slaughterhouses. Serovar Kennewicki is recognized as an animal pathogen [40], apparently adapted to pigs as maintenance host. Even though in Uruguay domestic pigs are not usually raised together with cattle, a forbidden practice in dairy farms, we should not rule out wild boars or other wild animals as potential hosts for this serovar, nor an endemic cycle in domestic cattle [2].

More information is needed to evaluate the prevalence of the serovars we have isolated in the whole country, and neighboring ones in South America. Furthermore, the virulence of these strains in relevant leptospirosis models will be important evidence that must be investigated, regarding pathogenicity (e.g. mortality in the hamster model) and renal colonization (e.g. in the bovine host). It is worth highlighting that we have isolated similar *Leptospira* species and sero-variants from chronic and acute cases in the field, as well as from dead animals from abattoirs, suggesting they represent a genuine sampling of the true population distribution of infectious *Leptospira* spp. in cattle. To be conclusive, an epidemiologic study with national geographic coverage is a necessary next step, as well as an in-depth molecular analysis of the *Leptospira* DNA recovered from PCR-positive urine samples that did not result in positive cultures.

At the individual animal level, and only considering herds with no recent history of vaccination (18 cases), the MAT technique correctly predicted the serogroup (Pomona) of 9 out of the 12 animals where *L*. *interrogans* strains were isolated (Table 2). In contrast, none of the 5 cases with *L*. *borgpetersenii* infections, nor the one from which a *L*. *noguchii* strain was isolated, presented detectable antibody titers using the diagnostic panel of reference available at the national diagnostics laboratory (DILAVE, MGAP). This is likely due to low sensitivity of the MAT, a known issue when it comes to host-acclimated serovars such as Hardjo in cattle [41]. The MAT did not identify any of the *L*. *noguchii* isolates, as these were not included within the reference antigen panel in the national diagnostics laboratories (DILAVE, Ministry of Livestock, Agriculture and Fishery). This finding is important, as *L*. *noguchii* is a recognized pathogenic species for animals and humans [33, 42]. However, when autochthonous *L*. *interrogans* serogroup Pomona, *L*. *borgpetersenii* serogroup Sejroe and representative serogroups of the *L*. *noguchii* strains were included for anti-*Leptospir*a antibodies titration by MAT, we did observe an increase of sensitivity: analyzing those herds with no history of recent vaccination, all the animals from which *L*. *borgpetersenii* strains were isolated showed reactivity against the local isolate, as it was also the case for an animal from which *L*. *noguchii* serogroup Pyrogenes was isolated (S5 Table).

As a consequence of this study, the inclusion of these native strains among the antigens for MAT diagnostics and seroprevalence epidemiologic studies, must be an immediate action. Such policies will be important to increase MAT-based diagnostics sensitivity and accuracy [43], and to improve the estimations of prevalence and incidence of bovine leptospirosis infection in the country. Furthermore, isolation and characterization of circulating *Leptospira* strains, are ongoing activities as a result of our multicentric consortium efforts. We anticipate that new variants and/or species may be discovered, achieving a more complete understanding of current diversity of *Leptospira* in South America.

A recent study of bovine *Leptospira* spp. isolates obtained from animals in slaughterhouses in Brazil, shows an important diversity in terms of species and serovars [14]. Libonati *et al*. report two *L*. *interrogans* strains belonging to serogroup Sejroe, and four different serogroups assigned to each of the other two *L*. *santarosai* and *L*. *noguchii* species identified. Our results now demonstrate a similar diversity of bovine isolates in terms of species and serovars. We have isolated *L*. *borgpetersenii* serogroup Sejroe strains, although so far, no *L*. *santarosai* isolates nor *L*. *interrogans* serogroup Sejroe have been recovered. Instead, we did isolate several strains of *L*. *interrogans* serogroup Pomona (presumptive serovar Kennewicki) and one Canicola (presumptive serovar Canicola). With regards to *L*. *noguchii*, the broad range of serogroups that we have detected seems to be a shared scenario with the situation in Brazil, with Autumnalis, Australis and Pyrogenes identified in both countries (additionally, serogroup Panama has also been identified in Brazil [32]). However, three *L*. *noguchii* isolates could not be classified in any serogroup, failing to agglutinate with the broad panel of reference antisera that was used. These results were confirmed in three different laboratories within our consortium, including the Paris center (WHO Collaborating Center and French reference laboratory for leptospirosis). In any case, these novel serogroups are distinct from the *L*. *noguchii* strains so far isolated in Brazil.

It does not escape our attention that most of the serovars that we are now reporting, are not included in the vaccines currently available to the farmers. Except for *L*. *borgpetersenii* serovar Hardjo and *L*. *interrogans* serovar Canicola, to the best of our knowledge neither serovar Kennewicki (*L*. *interrogans*) nor any of the *L*. *noguchii* serogroups/serovars that we identified, are being included in bacterin formulations that different companies produce and commercialize as bovine vaccines in South America (Table 2). Bacterins confer little or no cross-protection between serovars, hence the serovars that actually circulate in each region should be included to aim for efficacious vaccines [34]. Indeed, in our study we have obtained several isolates from one herd before and after vaccination. We will now perform closer analyses of naturally exposed herds, following up the effects of vaccination at the individual level. That current vaccines might have shifted the serovar profile of currently circulating *Leptospira* strains in Uruguay, is a plausible scenario. Proper bacterin vaccination should result in herd protection. We should have thus observed lower isolation rates from vaccinated herds, but we have not. Urine shedding of leptospires can be effectively controlled or significantly reduced in livestock, by using the correct bacterin formulations, according to recent studies with naturally exposed sheep herds [44] or with experimental vaccination/challenge approaches in cattle [45]. Significant reduction in bovine renal colonization and bacterial urinary shedding are achieved by vaccination with bacterins that include the infectious serovars [46], ultimately controlling endemic cycles of infection. Moreover, a systematic vaccination and surveillance program for pig and cattle leptospirosis in New Zealand, demonstrated a correlative dramatic decrease in the incidence, not only of the animal disease, but also of human leptospirosis [47]. Nevertheless, further research is needed to obtain long-lasting vaccination effects and complete protection against bacterial infection. Likely a protective cellular immune response is needed in the cattle model [46, 48, 49] to generate a highly efficacious vaccine against leptospirosis, and not only the humoral response triggered by killed-cell bacterins. The latter are also known to trigger a biased response towards the serovar-specific bacterial lipopolysaccharide antigen, T-independent with lack of memory response [50].

A more thorough understanding of leptospirosis epidemiology, including maintenance hosts and impact in livestock production, is essential to understand and design effective control strategies for this zoonosis. Efficacy studies with currently available vaccines for bovine leptospirosis in our region are also urgently needed. The assembly of this multicentric consortium (S1 Text) gathering the complementary expertise of several key research and governmental institutions in Uruguay, has made possible to obtain the first repository of *Leptospira* isolates in the public domain, most of them already typed in terms of species, serogroup and serovar. This is a major milestone in the way of controlling leptospirosis in Uruguay, with the associated far-reaching aim of reducing the risk for the human population.

## Supporting information

S1 Text Members of the “Grupo de Trabajo Interinstitucional de Leptospirosis” Consortium. (DOCX)

S1 Table Reference *Leptospira* strains used as antigens for antibody titration of bovine sera, by microscopic agglutination test. This panel is defined by the Uruguayan veterinarian health authorities (Ministry of Livestock, Agriculture and Fishery), and used for diagnostic purposes.
(DOCX)

S2 Table GenBank accession numbers for *secY* and *rrs* partial sequences obtained for all the *Leptospira* spp. isolates included in this work. (DOCX)

S3 Table Reference antisera used for serogroup determination by microscopic agglutination test. (DOCX)

S4 Table Effect of bovine urine in *L*. *borgpetersenii* serovar Hardjo cell viability. (DOCX)

S5 Table MAT of sera from individual animals from which pathogenic *Leptospira* strains were isolated, circumscribed to farms with no history of vaccination (see Table 2). Autochthonous *Leptospira* antigens are compared against the reference panel used by the national health agency.
(DOCX)

S1 Fig Representative profiles of Variable Number of Tandem Repeat (VNTR) analyses of *L*. *interrogans* and *L*. *borgpetersenii* autochthonous isolates. PCR amplifications of VNTR loci 4, 7, 10, Lb4 and Lb5, separated by agarose electrophoresis and ethidium bromide staining. Representative gels are included corresponding to: *L*. *interrogans* serogroup Pomona isolates IP1512014 and IP1512016 (lines 1 and 2, respectively); *L*. *interrogans* serogroup Canicola isolate IP1710049 (line 3) and *L*. *borgpetersenii* serogroup Sejroe isolates IP1506001, IP170430 and IP1708034 (lines 4, 5, 6, respectively). A PCR negative control is included in each gel, lanes labeled as (-). Molecular weight marker 100bp-ladders are included on side lanes, with a few reference sizes labeled in number of base pairs.
(DOCX)

S2 Fig Phylogeny of *Leptospira* spp. isolates based on *rrs* sequence analysis. Dendrogram using the neighbor-joining method (calculated using the Tamura-Neil model) plotting the relatedness of partial sequences of the 16S rRNA gene (*rrs*) including the 40 bovine isolates from Uruguay (blue labels) that we are now reporting. Sequences from 4 human isolates from Uruguay (green labels) were also included and plotted in comparison to 4 sequences corresponding to reference strains obtained elsewhere (red labels) and from different hosts, as indicated within parentheses. Isolates obtained in Uruguay are named according to their strain denomination as "IP" (Institut Pasteur Montevideo) or "IH" (Instituto de Higiene) followed by a 7- or 4-digit number, and after the vertical bar the GenBank accession number is reported for each one (S2 Table). Well separated phylogenetic clades correspond to different *Leptospira* species as indicated toward the right of the figure. The Patoc strain at the bottom of the panel belongs to the saprophytic species *L*. *biflexa*, and is included as a phylogenetic distance reference.
(DOCX)

## References

[1] Picardeau M. Virulence of the zoonotic agent of leptospirosis: still terra incognita? Nat Rev Microbiol. 2017;15(5):297–307. 10.1038/nrmicro.2017.5 28260786

[2] Ellis WA. Animal leptospirosis. Curr Top Microbiol Immunol. 2015;387:99–137. 10.1007/978-3-662-45059-8_6 25388134

[3] Haake DA, Levett PN. Leptospirosis in humans. Curr Top Microbiol Immunol. 2015;387:65–97. 10.1007/978-3-662-45059-8_5 25388133 PMC4442676

[4] Benschop J, Collins-Emerson J, Maskill A, O'Connor P, Tunbridge M, Yupiana Y, et al Leptospirosis in three workers on a dairy farm with unvaccinated cattle. N Z Med J. 2017;130(1462):102–108. 28934773

[5] Adler B. History of leptospirosis and leptospira. Curr Top Microbiol Immunol. 2015;387:1–9. 10.1007/978-3-662-45059-8_1 25388129

[6] Millan J, Cevidanes A, Chirife AD, Candela MG, Leon-Vizcaino L. Risk factors of Leptospira infection in Mediterranean periurban micromammals. Zoonoses Public Health. 2018;65(1):e79–e85. 10.1111/zph.12411 29058382

[7] Costa F, Hagan JE, Calcagno J, Kane M, Torgerson P, Martinez-Silveira MS, et al Global morbidity and mortality of leptospirosis: a systematic review. PLoS Negl Trop Dis. 2015;9(9):e0003898 10.1371/journal.pntd.0003898 26379143 PMC4574773

[8] Torgerson PR, Hagan JE, Costa F, Calcagno J, Kane M, Martinez-Silveira MS, et al Global burden of leptospirosis: estimated in terms of Disability Adjusted Life Years. PLoS Negl Trop Dis. 2015;9(10):e0004122 10.1371/journal.pntd.0004122 26431366 PMC4591975

[9] Caffarena RM CR, Cascelli ES, Martínez ES. Avances en leptospirosis en el Uruguay. Rev Urug Pat Clín Microbiol. 1971;9:186–194.

[10] Repiso MV, Gil A, Bañales PM, D'Anatro N, Fernández L, Guarino H, et al Prevalencia de las principales enfermedades infecciosas que afectan el comportamiento reproductivo en la ganadería de carne y caracterización de los establecimientos de cría del Uruguay. Veterinaria (Montevideo). 2005;40(157):5–28.

[11] Schelotto F, Hernandez E, Gonzalez S, Del Monte A, Ifran S, Flores K, et al A ten-year follow-up of human leptospirosis in Uruguay: an unresolved health problem. Rev Inst Med Trop Sao Paulo. 2012;54(2):69–75. 22499419 10.1590/s0036-46652012000200003

[12] Meny P, Menendez C, Quintero J, Hernandez E, Rios C, Balassiano IT, et al Characterization of *Leptospira* isolates from humans and the environment in Uruguay. Rev Inst Med Trop Sao Paulo. 2017;59:e79 10.1590/S1678-9946201759079 29267587 PMC5738764

[13] Meny P, Menendez C, Ashfield N, Rios C, Iglesias T, Quintero J, et al Leptospirosis in human groups at risk in Uruguay In: Society International Leptospirosis, editor. 10th International Leptispirosis Society Conference 2017 “Science for People”; Palmerston North, New Zealand 2017 p. 181.

[14] Libonati H, Pinto PS, Lilenbaum W. Seronegativity of bovines face to their own recovered leptospiral isolates. Microb Pathog. 2017;108:101–103. 10.1016/j.micpath.2017.05.001 28478204

[15] Chideroli RT, Pereira UP, Goncalves DD, Nakamura AY, Alfieri AA, Alfieri AF, et al Isolation and molecular characterization of *Leptospira borgpetersenii* serovar Hardjo strain Hardjobovis in the urine of naturally infected cattle in Brazil. Genet Mol Res. 2016;15(1): gmr8473. 10.4238/gmr.15018473 26909976

[16] Cosate MRV, Sakamoto T, de Oliveira Mendes TA, Moreira EC, Regis da Silva CG, Brasil B, et al Molecular typing of *Leptospira interrogans* serovar Hardjo isolates from leptospirosis outbreaks in Brazilian livestock. BMC Vet Res. 2017;13(1):177 10.1186/s12917-017-1081-9 28619055 PMC5471881

[17] Salgado M, Otto B, Moroni M, Sandoval E, Reinhardt G, Boqvist S, et al Isolation of *Leptospira interrogans* serovar Hardjoprajitno from a calf with clinical leptospirosis in Chile. BMC Vet Res. 2015;11:66 10.1186/s12917-015-0369-x 25888965 PMC4374366

[18] Myers DM, Jelambi F. Isolation and identification of *Leptospira* Hardjo from cattle in Argentina. Trop Geogr Med. 1975;27(1):63–70. 49113

[19] Director A, Penna B, Hamond C, Loureiro AP, Martins G, Medeiros MA, et al Isolation of *Leptospira interrogans* Hardjoprajitno from vaginal fluid of a clinically healthy ewe suggests potential for venereal transmission. J Med Microbiol. 2014;63(Pt 9):1234–1236. 10.1099/jmm.0.065466-0 24934563

[20] Carmona-Gasca CA, León Lara L, Castillo-Sánchez LO, Ramírez-Ortega JM, Ko A, Luna Palomera C, et al Detection of *Leptospira santarosai* and *L*. *kirschneri* in cattle: new isolates with potential impact in bovine production and public health. Vet Mex. 2011;42(4):277–288.

[21] Faine S, Adler B, Bolin C, Perolat P. *Leptospira* and leptospirosis 2nd ed Melbourne: MediSci; 1999. 272 p.

[22] Fouts DE, Matthias MA, Adhikarla H, Adler B, Amorim-Santos L, Berg DE, et al What makes a bacterial species pathogenic?: comparative genomic analysis of the genus *Leptospira*. PLoS Negl Trop Dis. 2016;10(2):e0004403 10.1371/journal.pntd.0004403 26890609 PMC4758666

[23] Galloway RL, Hoffmaster AR. Optimization of LipL32 PCR assay for increased sensitivity in diagnosing leptospirosis. Diagn Microbiol Infect Dis. 2015;82(3):199–200. 10.1016/j.diagmicrobio.2015.03.024 25912810 PMC6452440

[24] Hamond C, Martins G, Loureiro AP, Pestana C, Lawson-Ferreira R, Medeiros MA, et al Urinary PCR as an increasingly useful tool for an accurate diagnosis of leptospirosis in livestock. Vet Res Commun. 2014;38(1):81–85. 10.1007/s11259-013-9582-x 24222053

[25] Ahmed N, Devi SM, Valverde Mde L, Vijayachari P, Machang'u RS, Ellis WA, et al Multilocus sequence typing method for identification and genotypic classification of pathogenic *Leptospira* species. Ann Clin Microbiol Antimicrob. 2006;5:28 10.1186/1476-0711-5-28 17121682 PMC1664579

[26] Merien F, Amouriaux P, Perolat P, Baranton G, Saint Girons I. Polymerase chain reaction for detection of *Leptospira* spp. in clinical samples. J Clin Microbiol. 1992;30(9):2219–2224. 1400983 10.1128/jcm.30.9.2219-2224.1992 PMC265482

[27] Salaun L, Merien F, Gurianova S, Baranton G, Picardeau M. Application of multilocus variable-number tandem-repeat analysis for molecular typing of the agent of leptospirosis. J Clin Microbiol. 2006;44(11):3954–3962. 10.1128/JCM.00336-06 17088367 PMC1698352

[28] Ahmed A, Thaipadungpanit J, Boonsilp S, Wuthiekanun V, Nalam K, Spratt BG, et al Comparison of two multilocus sequence based genotyping schemes for *Leptospira* species. PLoS Negl Trop Dis. 2011;5(11):e1374 10.1371/journal.pntd.0001374 22087342 PMC3210738

[29] Jolley KA, Maiden MC. BIGSdb: Scalable analysis of bacterial genome variation at the population level. BMC Bioinformatics. 2010;11:595 10.1186/1471-2105-11-595 21143983 PMC3004885

[30] Chideroli RT, Goncalves DD, Suphoronski SA, Alfieri AF, Alfieri AA, de Oliveira AG, et al Culture strategies for isolation of fastidious *Leptospira* serovar Hardjo and molecular differentiation of genotypes Hardjobovis and Hardjoprajitno. Front Microbiol. 2017;8:2155 10.3389/fmicb.2017.02155 29163438 PMC5673650

[31] Hamond C, Pestana CP, Medeiros MA, Lilenbaum W. Genotyping of *Leptospira* directly in urine samples of cattle demonstrates a diversity of species and strains in Brazil. Epidemiol Infect. 2016;144(1):72–75. 10.1017/S0950268815001363 26076668 PMC9507312

[32] Martins G, Loureiro AP, Hamond C, Pinna MH, Bremont S, Bourhy P, et al First isolation of *Leptospira noguchii* serogroups Panama and Autumnalis from cattle. Epidemiol Infect. 2015;143(7):1538–1541. 10.1017/S0950268814002416 25185756 PMC9507209

[33] Silva ÉF, Cerqueira GM, Seyffert N, Seixas FK, Hartwig DD, Athanazio DA, et al *Leptospira noguchii* and human and animal leptospirosis, Southern Brazil. Emerg Infect Dis. 2009;15(4):621–623. 10.3201/eid1504.071669 19331754 PMC2671420

[34] Adler B, Moctezuma AD. *Leptospira* and leptospirosis. Vet Microbiol. 2010;140(3–4):287–296. 10.1016/j.vetmic.2009.03.012 19345023

[35] Miraglia F, de Morais ZM, Dellagostin OA, Seixas FK, Freitas JC, Zacarias FG, et al Molecular and serological characterization of *Leptospira interrogans* serovar Canicola isolated from dogs, swine, and bovine in Brazil. Trop Anim Health Prod. 2013;45(1):117–121. 10.1007/s11250-012-0181-6 22610538

[36] Levett PN. Leptospirosis. Clin Microbiol Rev. 2001;14(2):296–326. 10.1128/CMR.14.2.296-326.2001 11292640 PMC88975

[37] Arent ZJ, Gilmore C, San-Miguel Ayanz JM, Neyra LQ, Garcia-Pena FJ. Molecular epidemiology of *Leptospira* serogroup Pomona infections among wild and domestic animals in Spain. Ecohealth. 2017;14(1):48–57. 10.1007/s10393-017-1210-8 28213654

[38] Hathaway SC, Marshall RB, Little TW, Headlam SA, Winter PJ. Differentiation of reference strains of leptospires of the Pomona serogroup by cross-agglutination absorption and restriction endonuclease analysis. Res Vet Sci. 1985;39(2):145–150. 2999927

[39] Bolin CA, Zuerner RL. Correlation between DNA restriction fragment length polymorphisms in *Leptospira interrogans* serovar Pomona type Kennewicki and host animal source. J Clin Microbiol. 1996;34(2):424–425. 8789028 10.1128/jcm.34.2.424-425.1996 PMC228810

[40] Ellis WA. Leptospirosis In: Zimmerman J. J., Ramirez A., Schwartz K. J., Stevenson G. W., editors. Diseases of Swine. 10th ed Hoboken,NJ: Wiley-Blackwell; 2012 p. 770–778.

[41] Ellis WA. The diagnosis of leptospirosis in farm animals In: Ellis W.A., Little T.W.A., editors. The Present State of Leptospirosis Diagnosis and Control. Dordrecht, The Netherlands: Martinus Nijhoff Publishers; 1986 p. 13–24.

[42] Silva EF, Santos CS, Athanazio DA, Seyffert N, Seixas FK, Cerqueira GM, et al Characterization of virulence of *Leptospira* isolates in a hamster model. Vaccine. 2008;26(31):3892–3896. 10.1016/j.vaccine.2008.04.085 18547690 PMC2519131

[43] Pinto PS, Loureiro AP, Penna B, Lilenbaum W. Usage of *Leptospira* spp. local strains as antigens increases the sensitivity of the serodiagnosis of bovine leptospirosis. Acta Trop. 2015;149:163–167. 10.1016/j.actatropica.2015.05.008 25997883

[44] Vallee E, Ridler AL, Heuer C, Collins-Emerson JM, Benschop J, Wilson PR. Effectiveness of a commercial leptospiral vaccine on urinary shedding in naturally exposed sheep in New Zealand. Vaccine. 2017;35(9):1362–1368. 10.1016/j.vaccine.2016.04.037 27109564

[45] Bolin CA, Alt DP. Use of a monovalent leptospiral vaccine to prevent renal colonization and urinary shedding in cattle exposed to *Leptospira borgpetersenii* serovar Hardjo. Am J Vet Res. 2001;62(7):995–1000. 11453500 10.2460/ajvr.2001.62.995

[46] Zuerner RL, Alt DP, Palmer MV, Thacker TC, Olsen SC. A *Leptospira borgpetersenii* serovar Hardjo vaccine induces a Th1 response, activates NK cells, and reduces renal colonization. Clin Vaccine Immunol. 2011;18(4):684–691. 10.1128/CVI.00288-10 21288995 PMC3122574

[47] Thornley CN, Baker MG, Weinstein P, Maas EW. Changing epidemiology of human leptospirosis in New Zealand. Epidemiol Infect. 2002;128(1):29–36. 11895088 10.1017/s0950268801006392 PMC2869792

[48] Naiman BM, Alt D, Bolin CA, Zuerner R, Baldwin CL. Protective killed *Leptospira borgpetersenii* vaccine induces potent Th1 immunity comprising responses by CD4 and gammadelta T lymphocytes. Infect Immun. 2001;69(12):7550–7558. 10.1128/IAI.69.12.7550-7558.2001 11705932 PMC98846

[49] Naiman BM, Blumerman S, Alt D, Bolin CA, Brown R, Zuerner R, et al Evaluation of type 1 immune response in naive and vaccinated animals following challenge with *Leptospira borgpetersenii* serovar Hardjo: involvement of WC1(+) gammadelta and CD4 T cells. Infect Immun. 2002;70(11):6147–6157. 10.1128/IAI.70.11.6147-6157.2002 12379692 PMC130359

[50] Adler B. Vaccines against leptospirosis. Curr Top Microbiol Immunol. 2015;387:251–272. 10.1007/978-3-662-45059-8_10 25388138

