# Supplementary material for: Isolation of pathogenic Leptospira strains from naturally infected cattle in Uruguay reveals high serovar diversity, and uncovers a relevant risk for human leptospirosis
Source: PLoS Negl Trop Dis. 2018 Sep 13;12(9):e0006694. doi: 10.1371/journal.pntd.0006694 (PMC6136691; doi:10.1371/journal.pntd.0006694)
Supplement: S1 Text — (DOCX) [file pntd.0006694.s001.docx]

**Members of the “Grupo de Trabajo Interinstitucional de Leptospirosis” Consortium :**

*1- Plataforma Nacional de Investigacion en Salud Animal - Instituto Nacional de Investigación Agropecuaria (INIA)*

Maria Laura Casaux

Martín Fraga

Federico Giannitti

Melissa Macías-Rioseco

Cecilia Monesiglio

Yisell Perdomo

Franklin Riet-Correa

Caroline da Silva Silveira

*2- División de Laboratorios Veterinarios (DILAVE) - Ministerio de Ganadería, Agricultura y Pesca (MGAP)*

Gimena Avila

Natasha Barrandeguy

Carolina Briano

Florencia Buroni

Fernando Dutra

Maria Cristina Easton

Valentina Macchi

Alvaro Núnez

Rodolfo Rivero

Victor Rodriguez

Agustin Romero

Ximena Salaberry

Alejandra Suanes

*3- Departamento de Bacteriología y Virología, Instituto de Higiene, Universidad de la República (UdelaR)*

Natalia Ashfield

Tamara Iglesias

Clara Menendez

Paulina Meny

Jair Quintero

Cristina Rios

Felipe Schelotto

Gustavo Varela

*4- Unidad Mixta Pasteur + INIA, y Laboratorio de Microbiología Molecular y Estructural, Institut Pasteur de Montevideo*

Alejandro Buschiazzo

Joaquín Dalla Rizza

Camila Hamond

Juan Imelio

Nicole Larrieux

Cecilia Nieves

Marcos Nieves

Otto Pritsch

Fabiana San Martín

Felipe Trajtenberg

Leticia Zarantonelli
