# Supplementary material for: Isolation of pathogenic Leptospira strains from naturally infected cattle in Uruguay reveals high serovar diversity, and uncovers a relevant risk for human leptospirosis
Source: PLoS Negl Trop Dis. 2018 Sep 13;12(9):e0006694. doi: 10.1371/journal.pntd.0006694 (PMC6136691; doi:10.1371/journal.pntd.0006694)
Supplement: S1 Table — This panel is defined by the Uruguayan veterinarian health authorities (Ministry of Livestock, Agriculture and Fishery), and used for diagnostic purposes. (DOCX) [file pntd.0006694.s002.docx]

**S1 Table. Reference *Leptospira* strains used as antigens for antibody titration of bovine sera, by microscopic agglutination test.** This panel is defined by the Uruguayan veterinarian health authorities (Ministry of Livestock, Agriculture and Fishery), and used for diagnostic purposes.

| **Serogroup** | **Strain** | **Serovar** |
| --- | --- | --- |
| Pomona | Pomona | Pomona |
| Sejroe | Hardjo-Bovis | Hardjo |
| Sejroe | Hardjo-Prajitno | Hardjo |
| Sejroe | 3705 | Wolfii |
| Icterohaemorrhagiae | Ictero I | Icterohaemorrhagiae |
| Grippotyphosa | Moskva V | Grippotyphosa |
| Canicola | Hond Utrecht | Canicola |
