# Supplementary material for: Isolation of pathogenic Leptospira strains from naturally infected cattle in Uruguay reveals high serovar diversity, and uncovers a relevant risk for human leptospirosis
Source: PLoS Negl Trop Dis. 2018 Sep 13;12(9):e0006694. doi: 10.1371/journal.pntd.0006694 (PMC6136691; doi:10.1371/journal.pntd.0006694)
Supplement: S2 Table — (DOCX) [file pntd.0006694.s003.docx]

**S2 Table.** GenBank accession numbers for *secY* and *rrs* partial sequences obtained for all the *Leptospira* spp. isolates included in this work

| ***secY* sequence ID** | **GenBank Accession**  **number** | ***rrs s*equence ID** | **GenBank Accession number** |
| --- | --- | --- | --- |
| IP1506001 | MH325388 | IP1506001 | MH329307 |
| IP1507003 | MH325389 | IP1507003 | MH329308 |
| IP1509005 | MH325390 | IP1509005 | MH329309 |
| IP1509006 | MH325391 | IP1509006 | MH329310 |
| IP1509008 | MH325392 | IP1509008 | MH329311 |
| IP1509009 | MH325393 | IP1509009 | MH329312 |
| IP1509010 | MH325394 | IP1509010 | MH329313 |
| IP1512011 | MH325395 | IP1512011 | MH329314 |
| IP1512013 | MH325396 | IP1512013 | MH329315 |
| IP1512014 | MH325397 | IP1512014 | MH329316 |
| IP1512015 | MH325398 | IP1512015 | MH329317 |
| IP1512016 | MH325399 | IP1512016 | MH329318 |
| IP1512017 | MH325400 | IP1512017 | MH329319 |
| IP1603018 | MH325401 | IP1603018 | MH329320 |
| IP1605020 | MH325402 | IP1605020 | MH329321 |
| IP1605021 | MH325403 | IP1605021 | MH329322 |
| IP1609022 | MH325404 | IP1609022 | MH329323 |
| IP1610023 | MH325405 | IP1610023 | MH329324 |
| IP1611024 | MH325406 | IP1611024 | MH329325 |
| IP1611025 | MH325407 | IP1611025 | MH329326 |
| IP1611026 | MH325408 | IP1611026 | MH329327 |
| IP1703027 | MH325409 | IP1703027 | MH329328 |
| IP1703028 | MH325410 | IP1703028 | MH329329 |
| IP1703029 | MH325411 | IP1703029 | MH329330 |
| IP1704030 | MH325412 | IP1704030 | MH329331 |
| IP1704031 | MH325413 | IP1704031 | MH329332 |
| IP1705032 | MH325414 | IP1705032 | MH329333 |
| IP1708034 | MH325415 | IP1708034 | MH329334 |
| IP1708035 | MH325416 | IP1708035 | MH329335 |
| IP1708036 | MH325417 | IP1708036 | MH329336 |
| IP1709037 | MH325418 | IP1709037 | MH329337 |
| IP1709038 | MH325419 | IP1709038 | MH329338 |
| IP1710039 | MH325420 | IP1710039 | MH329339 |
| IP1710040 | MH325421 | IP1710040 | MH329340 |
| IP1710043 | MH325422 | IP1710043 | MH329341 |
| IP1710044 | MH325423 | IP1710044 | MH329342 |
| IP1710045 | MH325424 | IP1710045 | MH329343 |
| IP1710047 | MH325425 | IP1710047 | MH329344 |
| IP1711049 | MH325426 | IP1711049 | MH329345 |
| IP1712055 | MH325427 | IP1712055 | MH329346 |
| IH8783 | MH376290 | IH8783 | MH374851 |
| IH9790 | MH376291 | IH9790 | MH374852 |
| IH7829 | MH376292 | IH7829 | MH374853 |
| IH9597 | MH376293 | IH9597 | MH374854 |
