# Supplementary material for: Isolation of pathogenic Leptospira strains from naturally infected cattle in Uruguay reveals high serovar diversity, and uncovers a relevant risk for human leptospirosis
Source: PLoS Negl Trop Dis. 2018 Sep 13;12(9):e0006694. doi: 10.1371/journal.pntd.0006694 (PMC6136691; doi:10.1371/journal.pntd.0006694)
Supplement: S3 Table — (DOCX) [file pntd.0006694.s004.docx]

**S3 Table. Reference antisera used for serogroup determination by microscopic agglutination test.**

| **Antiserum #** | **Serogroup** | **Serovar** | **Strain** |
| --- | --- | --- | --- |
| 1 | Australis | Australis | Ballico |
| 2 | Autumnalis | Autumnalis | Akiyami A |
| 3 | Bataviae | Argentinensis | Peludo |
| 4 | Canicola | Portlandvere | MY 1039 |
| 5 | Ballum | Castellonis | Castellon 3 |
| 6 | Cynopteri | Cynopteri | 3522 C |
| 7 | Grippotyphosa | Grippotyphosa type Moskva | Moskva V |
| 8 | Sejroe | Hardjo type Bovis | Sponselee |
| 9 | Hebdomadis | Goiano | Bovino 131 |
| 10 | Icterohaemorrhagiae | Copenhageni | M 20 |
| 11 | Panama | Panama | CZ 214 |
| 12 | Semaranga | Patoc | Patoc I |
| 13 | Pomona | Proechimys | 1161 U |
| 14 | Pyrogenes | Pyrogenes | Salinem |
| 15 | Sejroe | Sejroe | M 84 |
| 16 | Tarassovi | Tarassovi | Perepelitsin |
| 17 | Icterohaemorrhagiae | Icterohaemorrhagiae | RGA |
| 18 | Celledoni | Celledoni | Celledoni |
| 19 | Djasiman | Djasiman | Djasiman |
| 20 | Mini | Mini | Sari |
| 21 | Sarmin | Rio | Rr 5 |
| 22 | Shermani | Shermani | 1342 K |
| 23 | Javanica | Javanica | Veldrat Batavia 46 |
| 24 | Louisiana | Louisiana | LSU 1945 |
