# Supplementary material for: Isolation of pathogenic Leptospira strains from naturally infected cattle in Uruguay reveals high serovar diversity, and uncovers a relevant risk for human leptospirosis
Source: PLoS Negl Trop Dis. 2018 Sep 13;12(9):e0006694. doi: 10.1371/journal.pntd.0006694 (PMC6136691; doi:10.1371/journal.pntd.0006694)
Supplement: S4 Table — (DOCX) [file pntd.0006694.s005.docx]

**S4 Table**. **Effect of bovine urine in *L. borgpetersenii* serovar Hardjo cell viability.**

| **Time of contact with urine before inoculation in EMJH media** | **Time of observation by DFM (weeks)** | **Evidence of growth by DFM with varying initial inocula (leptospira/mL)** | | | | | | | |
| --- | --- | --- | --- | --- | --- | --- | --- | --- | --- |
|  |  | **10^7^** | **10^6^** | **10^5^** | **10^4^** | **10^3^** | **10^2^** | **10** | **1** |
| 15 minutes | 1 | (+) | (+) | (+) | (-) | (-) | (-) | (-) | (-) |
|  | 2 | (+) | (+) | (+) | (+) | (-) | (-) | (-) | (-) |
|  | 3 | (+) | (+) | (+) | (+) | (+) | (+) | (-) | (-) |
|  | 4 | (+) | (+) | (+) | (+) | (+) | (+) | (+) | (+) |
|  | 5 | (+) | (+) | (+) | (+) | (+) | (+) | (+) | (+) |
| 1 hour | 1 | (+) | (+) | (-) | (-) | (-) | (-) | (-) | (-) |
|  | 2 | (+) | (+) | (+) | (+) | (-) | (-) | (-) | (-) |
|  | 3 | (+) | (+) | (+) | (+) | (+) | (+) | (-) | (-) |
|  | 4 | (+) | (+) | (+) | (+) | (+) | (+) | (+) | (+) |
|  | 5 | (+) | (+) | (+) | (+) | (+) | (+) | (+) | (+) |
| 2 hours | 1 | (+) | (+) | (-) | (-) | (-) | (-) | (-) | (-) |
|  | 2 | (+) | (+) | (+) | (-) | (-) | (-) | (-) | (-) |
|  | 3 | (+) | (+) | (+) | (+) | (+) | (-) | (-) | (-) |
|  | 4 | (+) | (+) | (+) | (+) | (+) | (+) | (+) | (-) |
|  | 5 | (+) | (+) | (+) | (+) | (+) | (+) | (+) | (+) |
| 6 hours | 1 | (+) | (+) | (-) | (-) | (-) | (-) | (-) | (-) |
|  | 2 | (+) | (+) | (+) | (-) | (-) | (-) | (-) | (-) |
|  | 3 | (+) | (+) | (+) | (+) | (-) | (-) | (-) | (-) |
|  | 4 | (+) | (+) | (+) | (+) | (+) | (+) | (-) | (-) |
|  | 5 | (+) | (+) | (+) | (+) | (+) | (+) | (+) | (-) |
