# Supplementary material for: Isolation of pathogenic Leptospira strains from naturally infected cattle in Uruguay reveals high serovar diversity, and uncovers a relevant risk for human leptospirosis
Source: PLoS Negl Trop Dis. 2018 Sep 13;12(9):e0006694. doi: 10.1371/journal.pntd.0006694 (PMC6136691; doi:10.1371/journal.pntd.0006694)
Supplement: S5 Table — Autochthonous Leptospira antigens are compared against the reference panel used by the national health agency. (DOCX) [file pntd.0006694.s006.docx]

**S5 Table.** MAT of sera from individual animals from which pathogenic *Leptospira* strains were isolated, circumscribed to farms with no history of vaccination (see Table 2). Autochthonous *Leptospira* antigens are compared against the reference panel used by the national health agency.

| **Strain #** | **Strain identification (serogroup and presumptive serovar)** | **Seroreactivity against reference strains (serogroup/titer)** | **Seroreactivity against autochtonous strains* (serogroup /titer)** |
| --- | --- | --- | --- |
| IP1507003 | *L. interrogans (*Pomona Kennewicki) | Pomona / 200 | Pomona / 6400 |
| IP1509008 | *L. interrogans (*Pomona Kennewicki) | nr | Pomona / 200 |
| IP1509009 | *L. interrogans (*Pomona Kennewicki) | Pomona / 400 | Pomona / 1600 |
| IP1509010 | *L. interrogans (*Pomona Kennewicki) | Pomona / 400 | Pomona / 6400 |
| IP1512011 | *L. interrogans (*Pomona Kennewicki) | nr | Pomona / 100 |
| IP1512014 | *L. interrogans (*Pomona Kennewicki) | Pomona / 400 | Pomona / 3200 |
| IP1710039 | *L. interrogans (*Pomona Kennewicki) | Pomona / 6400 | Pomona / 6400 |
| IP1710040 | *L. interrogans (*Pomona Kennewicki) | Pomona / 6400 Sejroe Hardjobovis / 3200 Sejroe Hardjoprajitno / 1600 Sejroe Wolffii / 1600 | Pomona / 6400 |
| IP1710043 | *L. interrogans (*Pomona Kennewicki) | Pomona / 3200 Sejroe Hardjobovis / 800 | Pomona / 1600 |
| IP1710044 | *L. interrogans (*Pomona Kennewicki) | Pomona / 3200 Serjoe Hardjobovis / 3200 Serjoe Hardjoprajitno / 800 | Pomona / 3200 |
| IP1710045 | *L. interrogans (*Pomona Kennewicki) | Pomona / 6400 | Pomona / 6400 |
| IP1710047 | *L. interrogans (*Pomona Kennewicki) | nr | Pomona / 100 |
| IP1506001 | *L. borgpetersenii* (Sejroe Hardjo) | Pomona / 400 | Sejroe / 200 |
| IP1509005 | *L. borgpetersenii* (Sejroe Hardjo) | nr | Sejroe / 400 |
| IP1509006 | *L. borgpetersenii* (Sejroe Hardjo) | nr | Sejroe / 800 |
| IP1512013 | *L. borgpetersenii* (Sejroe Hardjo) | nr | Sejroe / 400 |
| IP1708034 | *L. borgpetersenii* (Sejroe Hardjo) | nr | Sejroe / 400 |
| IP1605021 | *L. noguchii* (Pyrogenes) | nr | Pyrogenes / 100 |

***** The panel of autochthonous strains used as antigens included representative local isolates belonging to homologous serogroups Pomona, Sejroe and Pyrogenes; **nr**: non-reactive (below cutoff titer ≥100)
