# Supplementary material for: Isolation of pathogenic Leptospira strains from naturally infected cattle in Uruguay reveals high serovar diversity, and uncovers a relevant risk for human leptospirosis
Source: PLoS Negl Trop Dis. 2018 Sep 13;12(9):e0006694. doi: 10.1371/journal.pntd.0006694 (PMC6136691; doi:10.1371/journal.pntd.0006694)
Supplement: S1 Fig — PCR amplifications of VNTR loci 4, 7, 10, Lb4 and Lb5, separated by agarose electrophoresis and ethidium bromide staining. Representative gels are included corresponding to: L. interrogans serogroup Pomona isolates IP1512014 and IP1512016 (lines 1 and 2, respectively); L. interrogans serogroup Canicola isolate IP1710049 (line 3) and L. borgpetersenii serogroup Sejroe isolates IP1506001, IP170430 and IP1708034 (lines 4, 5, 6, respectively). A PCR negative control is included in each gel, lanes labeled as (-). Molecular weight marker 100bp-ladders are included on side lanes, with a few reference sizes labeled in number of base pairs. (DOCX) [file pntd.0006694.s007.docx]

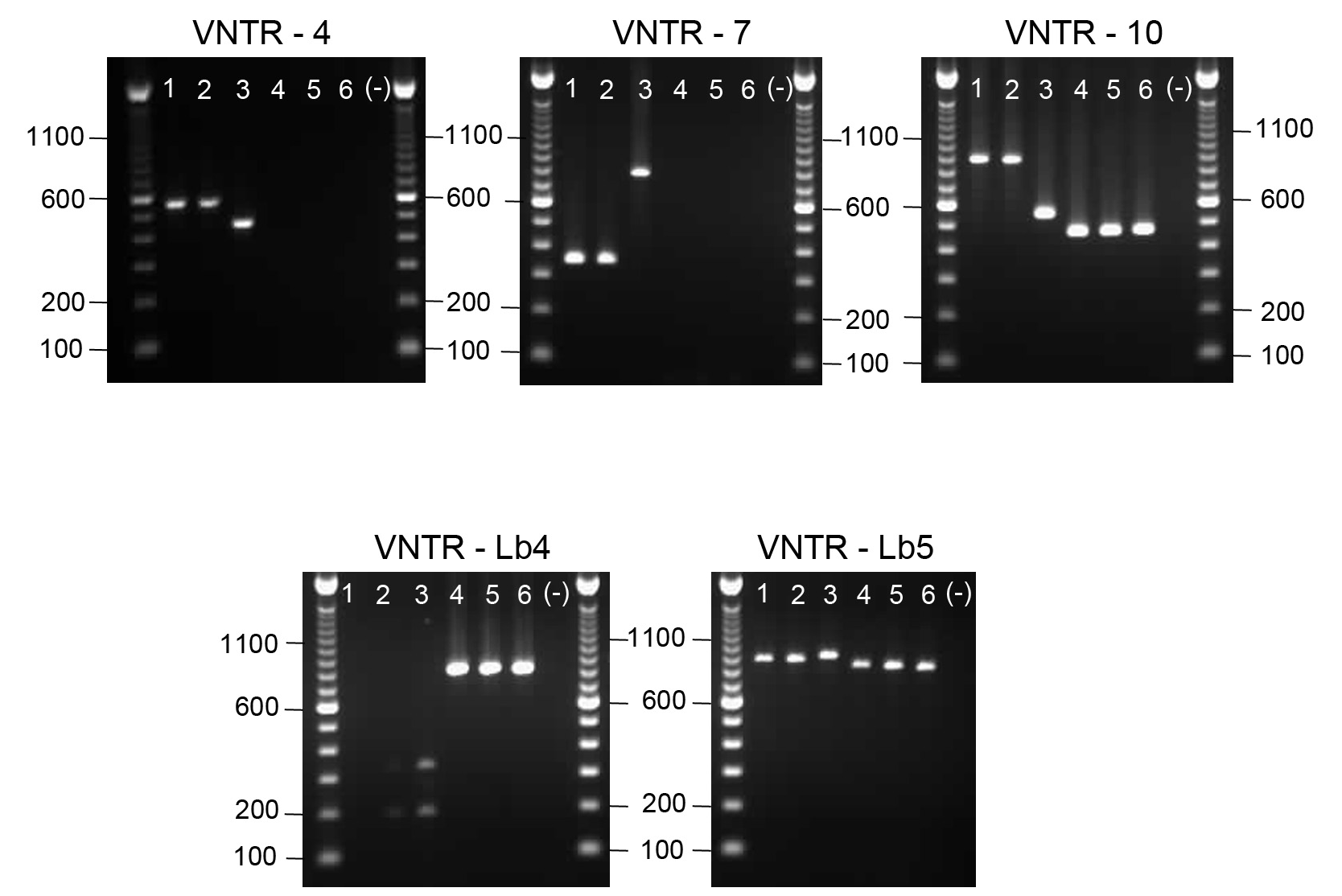


**S1 Figure. Representative profiles of Variable Number of Tandem Repeat (VNTR) analyses of *L. interrogans* and *L. borgpetersenii* autochthonous isolates.** PCR amplifications of VNTR loci 4, 7, 10, Lb4 and Lb5, separated by agarose electrophoresis and ethidium bromide staining. Representative gels are included corresponding to: *L. interrogans* serogroup Pomona isolates IP1512014 and IP1512016 (lines 1 and 2, respectively); *L. interrogans* serogroup Canicola isolate IP1710049 (line 3) and *L. borgpetersenii* serogroup Sejroe isolates IP1506001, IP170430 and IP1708034 (lines 4, 5, 6, respectively). A PCR negative control is included in each gel, lanes labeled as (-). Molecular weight marker 100bp-ladders are included on side lanes, with a few reference sizes labeled in number of base pairs.
