# Supplementary material for: Isolation of pathogenic Leptospira strains from naturally infected cattle in Uruguay reveals high serovar diversity, and uncovers a relevant risk for human leptospirosis
Source: PLoS Negl Trop Dis. 2018 Sep 13;12(9):e0006694. doi: 10.1371/journal.pntd.0006694 (PMC6136691; doi:10.1371/journal.pntd.0006694)
Supplement: S2 Fig — Dendrogram using the neighbor-joining method (calculated using the Tamura-Neil model) plotting the relatedness of partial sequences of the 16S rRNA gene (rrs) including the 40 bovine isolates from Uruguay (blue labels) that we are now reporting. Sequences from 4 human isolates from Uruguay (green labels) were also included and plotted in comparison to 4 sequences corresponding to reference strains obtained elsewhere (red labels) and from different hosts, as indicated within parentheses. Isolates obtained in Uruguay are named according to their strain denomination as "IP" (Institut Pasteur Montevideo) or "IH" (Instituto de Higiene) followed by a 7- or 4-digit number, and after the vertical bar the GenBank accession number is reported for each one (S2 Table). Well separated phylogenetic clades correspond to different Leptospira species as indicated toward the right of the figure. The Patoc strain at the bottom of the panel belongs to the saprophytic species L. biflexa, and is included as a phylogenetic distance reference. (DOCX) [file pntd.0006694.s008.docx]

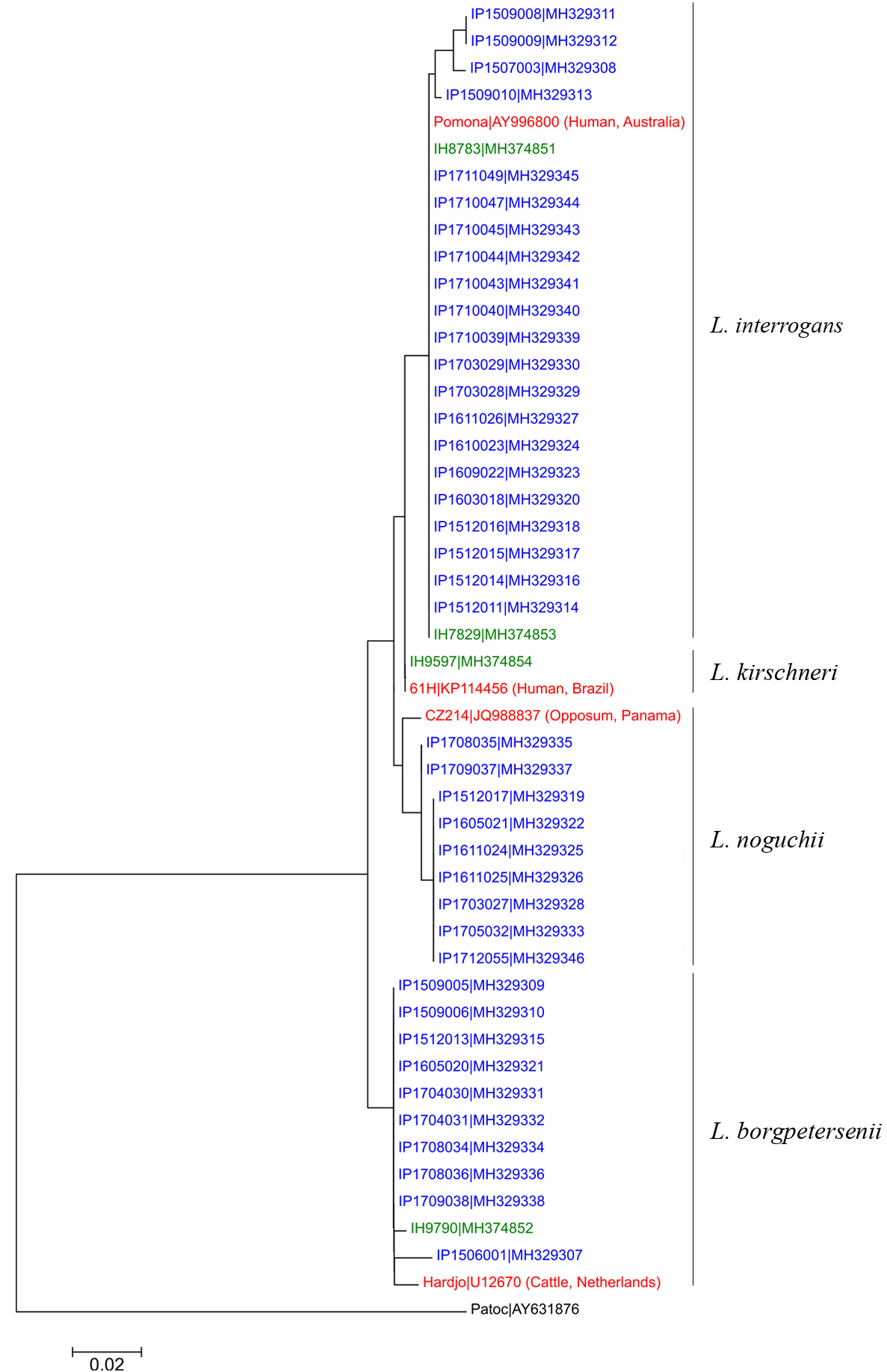


**S2 Figure. Phylogeny of *Leptospira* spp. isolates based on *rrs* sequence analysis.** Phylogenetic tree inferred using the Neighbor-Joining method. The tree is drawn to scale, with branch lengths in the same units as those of the evolutionary distances used to infer the phylogenetic tree. The evolutionary distances were computed using the Tamura-Nei method, in the units of the number of base substitutions per site. The relatedness of 49 partial sequences of the 16S rRNA gene (*rrs*) have been compared, including the 40 bovine isolates from Uruguay (blue labels) that we are now reporting. Sequences from 4 human isolates from Uruguay (green labels) were also included and plotted in comparison to 4 sequences corresponding to reference strains obtained elsewhere (red labels) and from different hosts, as indicated within parentheses. Isolates obtained in Uruguay are named according to their strain denomination as "IP" (Institut Pasteur Montevideo) or "IH" (Instituto de Higiene) followed by a 7- or 4-digit number. GenBank accession numbers are reported within each strain name after the vertical bar. Well separated phylogenetic clades correspond to different *Leptospira* species as indicated toward the right of the figure. The Patoc strain at the bottom of the panel belongs to the saprophytic species *L. biflexa*.
